# Gastrointestinal cancer: molecular pathogenesis and targeted therapy

**DOI:** 10.1186/s43556-025-00361-9

**Published:** 2025-12-09

**Authors:** Yang Jin, Xiaobo He, Yanfeng Wu

**Affiliations:** 1 College of Basic Medical Sciences, Naval Medical University, Shanghai, 200433 People’s Republic of China; 2 National Key Laboratory of Immunity and Inflammation & Institute of Immunology, College of Basic Medical Sciences, Naval Medical University, Shanghai, 200433 People’s Republic of China

**Keywords:** Gastrointestinal tumors, Immunotherapy, Targeted Therapy, Signal Transduction

## Abstract

Gastrointestinal (GI) cancers pose a significant global health burden, driven by complex molecular alterations and microenvironmental interactions. Advances in molecular pathogenesis have elucidated recurrent driver gene mutations, such as *KRAS*, *TP53*, and *APC*, alongside dysregulated signaling pathways including Wnt, RAS-MAPK, and PI3K-AKT, which collectively underpin tumor initiation and progression. Complementing genetic changes, epigenetic alterations—such as DNA hypermethylation, histone modifications, and regulatory non-coding RNAs—further contribute to malignant evolution by reshaping chromatin architecture and gene expression. These mechanisms not only promote uncontrolled proliferation but also reinforce therapeutic resistance by dynamically modifying the tumor microenvironment (TME). Molecular subtyping efforts, including The Cancer Genome Atlas (TCGA) classification for gastric cancer (GC) and the Consensus Molecular Subtypes (CMS) for colorectal cancer (CRC), have delineated disease heterogeneity, revealing distinct pathogenic pathways and enabling refined prognostic stratification. Such insights provide the biological rationale for diagnostic techniques and targeted interventions. For instance, anti-EGFR and anti-VEGF monoclonal antibodies disrupt oncogenic signaling and tumor angiogenesis, respectively, and have demonstrated substantial clinical efficacy in selected patient populations. In parallel, immunotherapy has emerged as a transformative modality in oncology. Immune checkpoint inhibitors targeting PD-1/PD-L1 and CTLA-4 reinvigorate antitumor immunity and have reshaped standard-of-care protocols for several GI malignancies. Beyond conventional immunotherapies, innovative strategies such as CAR-T cell therapy and neoantigen-based vaccines are being actively investigated. These approaches aim to overcome immune evasion mechanisms and enhance tumor-specific targeting, offering promise for patients with resistant or advanced disease. This review comprehensively analyzes the evolving molecular landscape of GI cancers and the corresponding development of targeted and immunotherapeutic agents. It highlights a balanced integration of mechanistic discovery and clinical translation, underscoring their synergistic roles in advancing precision oncology and improving survival outcomes.

## Introduction

Gastrointestinal (GI) cancers represent a significant global health challenge, with approximately 4.8 million new cases and 3.4 million deaths annually, making them one of the leading causes of cancer-related mortality. A deeper understanding of their underlying molecular mechanisms, including driver genes, signaling pathways, and the tumor microenvironment (TME), is therefore crucial for laying the foundation to develop more effective therapeutic regimens.

The evolution of precision medicine in GI cancers demonstrates distinct developmental phases. Early research focused on driver genes (e.g., *KRAS*, *TP53*, *APC*) and core signaling pathways (e.g., Wnt, RAS-MAPK), which established the theoretical foundation for targeted therapy and led to the development of treatments such as anti-EGFR and anti-VEGF therapies [1–3]. Subsequently, molecular subtyping efforts like The Cancer Genome Atlas (TCGA) and Consensus Molecular Subtypes (CMS) classified tumors into subtypes such as EBV-positive, microsatellite instability-high (MSI-H), chromosomally unstable (CIN), and genomically stable (GS), systematically elucidating disease heterogeneity and guiding the shift toward “same disease, different treatment” strategies [4, 5]. The breakthrough in 2015 regarding the MSI-H/mismatch repair-deficient (dMMR) mechanism served as a critical link between genotype and the immune microenvironment, expanding the scope of precision medicine from targeting tumor cells to immune modulation [6]. More recently, advances in epigenetic mechanisms (e.g., *IDH1* mutations) and the exploration of rare targets (e.g., *NTRK* fusions, CLDN18.2) signify the ongoing maturation and comprehensive refinement of precision therapy frameworks [7, 8].

This review systematically surveys the molecular basis and targeted therapeutic strategies for GI cancers. It elucidates their molecular pathogenesis, including the genetic and epigenetic alterations that drive tumorigenesis, signaling pathway aberrations, and the regulatory role of the TME. Furthermore, it discusses current diagnostic methods, including the role of early detection biomarkers and advanced imaging techniques in improving diagnostic accuracy. Regarding therapeutic approaches, this review critically evaluates the clinical efficacy and limitations of existing targeted therapies (such as monoclonal antibodies, small molecule inhibitors, and immunotherapies) and explores emerging treatment strategies, including mesenchymal stem cell-derived exosomes, genome-driven personalized medicine, and combination therapies. Through this systematic overview, this article aims to provide a theoretical basis and outline future research directions for the mechanistic study, clinical management, and translational practice in the field of GI cancers.

## Molecular pathogenesis of GI cancer

The cell cycle is a tightly regulated process that orchestrates cell growth, DNA replication, and division. It relies on a series of checkpoints (G1/S, G2/M, and spindle assembly checkpoint) to ensure genomic stability and prevent the propagation of damaged DNA [9]. These checkpoints act as molecular gatekeepers, halting cell cycle progression in response to DNA damage or replication errors. However, in cancer, including GI malignancies, the disruption of these checkpoints is a fundamental event that drives uncontrolled proliferation and genomic instability.

The G1/S checkpoint is particularly critical, as it determines whether a cell commits to DNA replication. This checkpoint is regulated by tumor suppressor genes such as *TP53* and *RB1*, which act as brakes on cell cycle progression under conditions of genomic stress. Similarly, the G2/M checkpoint ensures that DNA is fully repaired before mitosis, with proteins such as ATM, CHK2, and BRCA1 playing essential roles. Dysregulation in these pathways contributes to chromosomal instability, a hallmark of cancer progression.

The progression of the cell cycle is driven by cyclin-dependent kinases (CDKs) and their regulatory partners, cyclins. For example, Cyclin D1 and CDK4/6 promote the transition from G1 to S phase by phosphorylating the RB1 protein, effectively braking the cell cycle progression. In GI cancers, overexpression of Cyclin D1 or hyperactivation of CDK4/6 bypasses the G1/S checkpoint, leading to uncontrolled proliferation.

**Fig. 1:**
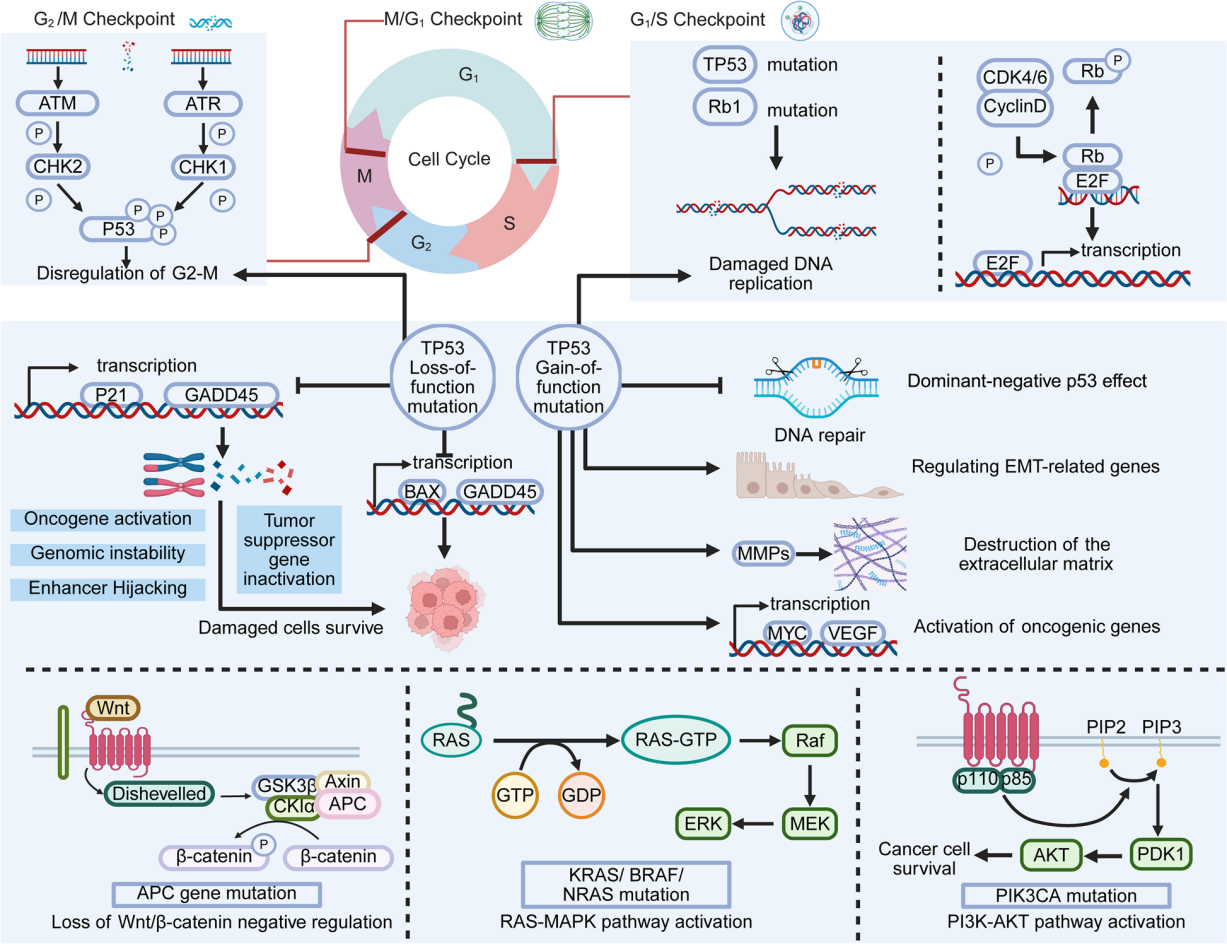
Genetic Mechanisms Underlying Gastrointestinal Cancer Progression. Dysregulation of cell cycle checkpoints (G1/S, M/G2, and G2/M) contributes to genomic instability and tumor suppressor gene inactivation. Mutations in key genes such as *TP53* lead to loss-of-function (LOF) effects that enable damaged cells to survive or gain-of-function effects that promote oncogenic transcription, extracellular matrix destruction, and epithelial-mesenchymal transition (EMT). Additional mutations in APC, KRAS/BRAF/NRAS, and PIK3CA activate oncogenic pathways, including Wnt/β-catenin signaling, RAS-MAPK signaling, and PI3K-AKT signaling, driving tumorigenesis and cancer cell survival (figure was created with Biorender.com)

In brief, the dysregulation of cell cycle checkpoints and the aberrant activation of cyclin-CDK complexes are central to the molecular pathogenesis of GI cancers. These processes are frequently driven by mutations in key genes such as *TP53*, *KRAS*, and *APC*, which not only disrupt normal cell cycle control but also initiate downstream oncogenic signaling pathways (Fig. 1). In detail, *KRAS* mutations drive constitutive activation of signaling axes such as MAPK and PI3K-AKT, which collaboratively upregulate and stabilize key G1/S checkpoint regulators, particularly Cyclin D1, at both the transcriptional and translational levels, thereby relieving the inhibition on pRb [10]. The following sections will explore these high-frequency genetic alterations and their roles in tumorigenesis.

### Genetic alterations

#### Esophageal squamous cell carcinoma

With the exceedingly high mutation rate in esophageal squamous cell carcinoma (over 80%) [11], inactivation of the tumor suppressor gene *TP53* is a hallmark event in the development of most GI cancers, representing the single most frequently mutated gene in this group of malignancies. As the “guardian of the genome”, wild-type p53 responds to cellular stress by inducing cell cycle arrest, DNA repair, or apoptosis. The mutational landscape of *TP53* is diverse, encompassing truncating (nonsense and frameshift) mutations that lead to a complete loss-of-function (LOF), as well as missense mutations predominantly located in the DNA-binding domain [12]. However, it has been well established that different *TP53* mutations lead to distinct functional consequences. While truncating mutations result in the absence of the p53 protein, a subset of missense mutations (e.g., R175H, R248Q, R273H) confer a neomorphic, oncogenic gain-of-function (GOF) [13]. These GOF mutants not only inhibit wild-type tumor-suppressive activities through a dominant-negative effect but also actively promote tumor progression, invasion, metastasis, and therapeutic resistance [14]. Because, GOF in *TP53* hijack and alter the expression of various DNA repair genes (such as *MRE11* and *RAD51*) and disrupt normal replication processes, thereby exacerbating genomic instability [15]. Furthermore, these mutants promote chemotherapy resistance by activating proto-oncogenes like *c-MYC* and co-opt chromatin regulatory pathways to reprogram the epigenetic landscape [16, 17]. From the perspective of metabolic reprogramming, the expression of glucose transporters such as GLUT1 is also upregulated, enhancing the Warburg effect to provide sustained energy and biosynthetic precursors for the malignant proliferation of tumor cells [18]. Patients with tumors harboring GOF mutations often exhibit a more aggressive phenotype and worse outcomes compared to those with LOF mutations or wild-type *TP53* [19]. It is evident that the loss of p53 function is a critical, often late-stage event that is necessary for malignant progression, but it typically acts in concert with other oncogenic alterations for full-blown cancer development [20]. Here, *TP53* loss-of-function mutations serve as a critical driver in the malignant progression of gastrointestinal tumors [21]. Their increased frequency in advanced stages stems from clonal selection under evolutionary pressure, where genomic instability and therapeutic stress favor the survival of *TP53*-deficient clones [22]. By disrupting cell cycle checkpoints, apoptosis induction, and DNA repair mechanisms, these mutations promote genomic chaos, therapy resistance, and metastatic competence [23]. Ultimately, the inactivation of this “genome guardian” equips tumor cells with the ultimate survival advantage to withstand stress, accelerate evolution, and overcome microenvironmental constraints.

In detail, in colorectal cancer, *TP53* mutations primarily occur during the transition from benign adenoma to invasive carcinoma, resulting in a very high mutation rate (approximately 60%) in invasive carcinoma (late-stage adenocarcinoma), while being relatively rare in the adenoma stage [24]. Regarding gastric cancer, the overall incidence of *TP53* mutations is slightly lower (33%), and the incidence of *TP53* mutations in advanced stages (III/IV) is significantly higher than in early stages (I/II) [25]. In pancreatic cancer (PC), *TP53* mutation is also a relatively late genetic event, typically appearing at the high-grade pancreatic intraepithelial neoplasia (PanIN-3, i.e., carcinoma in situ) stage [26].

#### Cholangiocarcinoma

The molecular profile of intrahepatic cholangiocarcinoma (iCCA) is notably different from other GI cancers. Furthermore, the notable incidence of *FGFR2* alterations and *IDH* mutations, coupled with the advancing development of targeted therapeutics against these molecular abnormalities, represents an area of growing clinical importance. Regarding the oncogenic mechanism of *FGFR2* alterations, FGFR2 functions as a receptor tyrosine kinase. Critically, the loss of its C-terminal inhibitory domain, often resulting from deletions in the terminal exon, leads to the formation of constitutively active truncated FGFR2 isoforms. These variants dimerize and signal ligand-independently, driving uncontrolled proliferation and tumor survival, inducing conformational changes in disulfide bonds via modified cysteine residues, thereby promoting receptor dimerization and subsequent FGFR2 activation [27–29]. More importantly, *FGFR2* fusions are identified in 10%–20% of patients with iCCA. Consequently, for iCCA patients, clinical guidelines routinely recommend the use of next-generation sequencing (NGS) or fluorescence in situ hybridization (FISH) to identify individuals eligible for FGFR2-targeted therapy. The corresponding targeted agents, primarily pemigatinib and infigratinib, will be detailed in Sect. “Monoclonal antibodies and small molecular drugs”. In CCA, *IDH* mutations are also detected in approximately 20% of patients, with the *IDH1* R132C variant being the most prevalent [7, 30]. Mechanistically, GOF mutations in the isocitrate dehydrogenase gene lead to the aberrant production and accumulation of the oncometabolite 2-hydroxyglutarate (2-HG), which disrupts cellular epigenetic regulation and impairs differentiation, thereby promoting tumorigenesis. Given the rarity of *IDH* mutations in other gastrointestinal malignancies, they are regarded as a relatively specific molecular marker for iCCA. Testing for *IDH1/2* mutations is considered a standard component of the diagnostic workup for confirmed iCCA cases. Representative targeted therapies for *IDH*-mutant tumors, such as ivosidenib, will also be discussed in Sect. “Monoclonal antibodies and small molecular drugs”.

#### Colorectal cancer

Colorectal carcinogenesis follows a well-defined genetic sequence. An early, the genetic landscape of GI cancers is defined by alterations in key signaling pathways. Loss-of-function mutations in the tumor suppressor adenomatous polyposis coli (*APC*), a negative regulator of the Wnt/β-catenin pathway, represent an early, gatekeeping event, occurring in over 80% of sporadic tumors [24]. Familial adenomatous polyposis (FAP) results from germline *APC* mutations, predisposing individuals to thousands of colonic polyps [31]. This is followed by activating mutations in *TP53* (~ 60%), which is often a late event facilitating malignant progression. Activating mutations in the RAS-MAPK pathway, primarily in *KRAS* (~ 41%) and to a lesser extent *BRAF* or *NRAS*, are also critical drivers [24]. Mutations in *PIK3CA*, which activate the PI3K-AKT signaling cascade, are found in 12% and 18% of GC and CRCs respectively [4, 24].

Furthermore, a significant subset of GI cancers arises from inherited germline mutations. Lynch syndrome, the most common hereditary CRC syndrome, is caused by germline mutations in DNA mismatch repair (MMR) genes such as *MLH1*, *MSH2*, *MSH6*, or *PMS2*, leading to a microsatellite instability (MSI) phenotype [32, 33].

#### Gastric cancer

In GC, *TP53* mutations are present in approximately 50% of cases [4]. Beyond *TP53*, the disease is characterized by significant heterogeneity. Key alterations include mutations in *PIK3CA* (12%) activating the PI3K-AKT pathway, and amplifications of receptor tyrosine kinases such as ERBB2 (HER2), EGFR, and MET, which serve as critical therapeutic targets [3, 4, 24, 34]. The *ERBB2* (also known as *HER2*) gene is amplified in 11–16% of gastroesophageal adenocarcinoma, with 20% overexpression [35], serving as a well-established biomarker for anti-HER2 targeted therapies, such as trastuzumab [36]. Furthermore, *FGFR2* alterations, including amplifications (5–10%) and mutations (~ 4%), represent additional oncogenic drivers in GC [37, 38]. In diffuse-type gastric cancer, particularly in its hereditary form (HDGC), germline mutations in the *CDH1* gene represent the primary pathogenic mechanism. Loss of function in its encoded protein, E-cadherin, leads to critical defects in cellular adhesion [39]. The lifetime cumulative risk of *DGC* in *CDH1* mutation carriers by age 80 to be 70% (95% CI, 59%-80%) in men and 56% (95% CI, 44%-69%) in women [40].

#### ancreatic cancer

 Pancreatic ductal adenocarcinoma is molecularly driven by *KRAS* mutations observed in approximately 93% of cases, most frequently at the G12D codon [41]. These mutations lock the RAS protein in a constitutively active GTP-bound state, leading to ligand-independent activation of downstream effector pathways, including RAF-MEK-ERK and PI3K-AKT, thereby promoting tumor cell proliferation and survival.

The primary function of homologous recombination is to repair DNA double-strand breaks and interstrand cross-links, and it can also restore stalled or broken replication forks [42]. The pathogenesis of a range of PC is associated with mutations in genes related to this pathway. The accurate repair of DNA double-strand breaks via homologous recombination is orchestrated by the BRCA1 and BRCA2 proteins. BRCA1 acts as a master regulator, responsible for detecting the lesion, recruiting the repair machinery, and determining the repair pathway. BRCA2 is then entrusted with the critical task of loading RAD51 onto the DNA to perform strand invasion, a step that utilizes the sister chromatid to faithfully restore the original DNA sequence [43, 44]. This essential repair mechanism is compromised in the 4%–7% of pancreatic cancers harboring germline mutations in these genes [45–47]. In terms of targeted therapy, poly ADP-ribose polymerase (PARP) inhibitors have demonstrated superior efficacy in pancreatic cancer and hepatobiliary cancer patients carrying *BRCA1/2* mutations [45, 48]. Moreover, patients with *BRCA1/2* mutations show higher response rates and more significant survival benefits when treated with platinum-based chemotherapy regimens (e.g., FOLFIRINOX) [49]. Notably, the prevalence of homologous recombination deficiency (HRD) in PC ranged between 14.5% and 16.5% [50]. In contrast, the incidence of HRD is particularly low in colorectal cancer, which may be attributable to the high microsatellite instability already driving tumorigenesis in this malignancy. Overall, for gastrointestinal tumors closely linked to homologous recombination deficiency, especially pancreatic cancer, attention should be given to the use of PARP inhibitors and platinum-based chemotherapy regimens.

#### GEP-NENs

Neuroendocrine neoplasms (NENs), tumors derived from neuroendocrine cells, are most frequently located in the gastroenteropancreatic system (GEP-NENs, 55%-70%), with rising incidence in the US (6.98/100,000 in 2012) and an annual incidence of 1.33–2.33/100,000 in Europe [51–53]. GEP-NENs exhibit significant genetic heterogeneity, with distinct mutational profiles based on the primary site: pancreatic NENs most frequently harbor mutations in *MEN1* [54, 55], followed by alterations in *DAXX *(death domain associated protein) or *ATRX* (alpha thalassemia/mental retardation syndrome X-Linked) and genes in the mTOR pathway [56, 57]; small intestinal NENs typically lack these pancreatic-associated mutations and are instead closely linked to *CDKN1B* (cyclin dependent kinase inhibitor 1B/2C) mutations [58]; while colorectal NENs often involve classic tumor suppressor genes such as *TP53* and *APC*, showing a partial overlap with the genetic landscape of colorectal adenocarcinoma [59].

#### Other GI cancers

Meanwhile, gastrointestinal stromal tumors (GISTs) are primarily characterized by *KIT* mutations (approximately 80%), particularly in the juxtamembrane domain (e.g., exon 11), which result in ligand-independent dimerization and constitutive activation of the receptor tyrosine kinase. This molecular mechanism underlies the exceptional sensitivity of GIST to tyrosine kinase inhibitors such as imatinib, establishing it as a paradigm for molecularly targeted therapy in solid tumors. In hepatocellular carcinoma, *TERT* promoter mutations represent the most prevalent genetic alteration (approximately 60%), which enhance telomerase reverse transcriptase expression and confer cellular immortality by maintaining telomere length.

### Epigenetic alterations

**Fig. 2:**
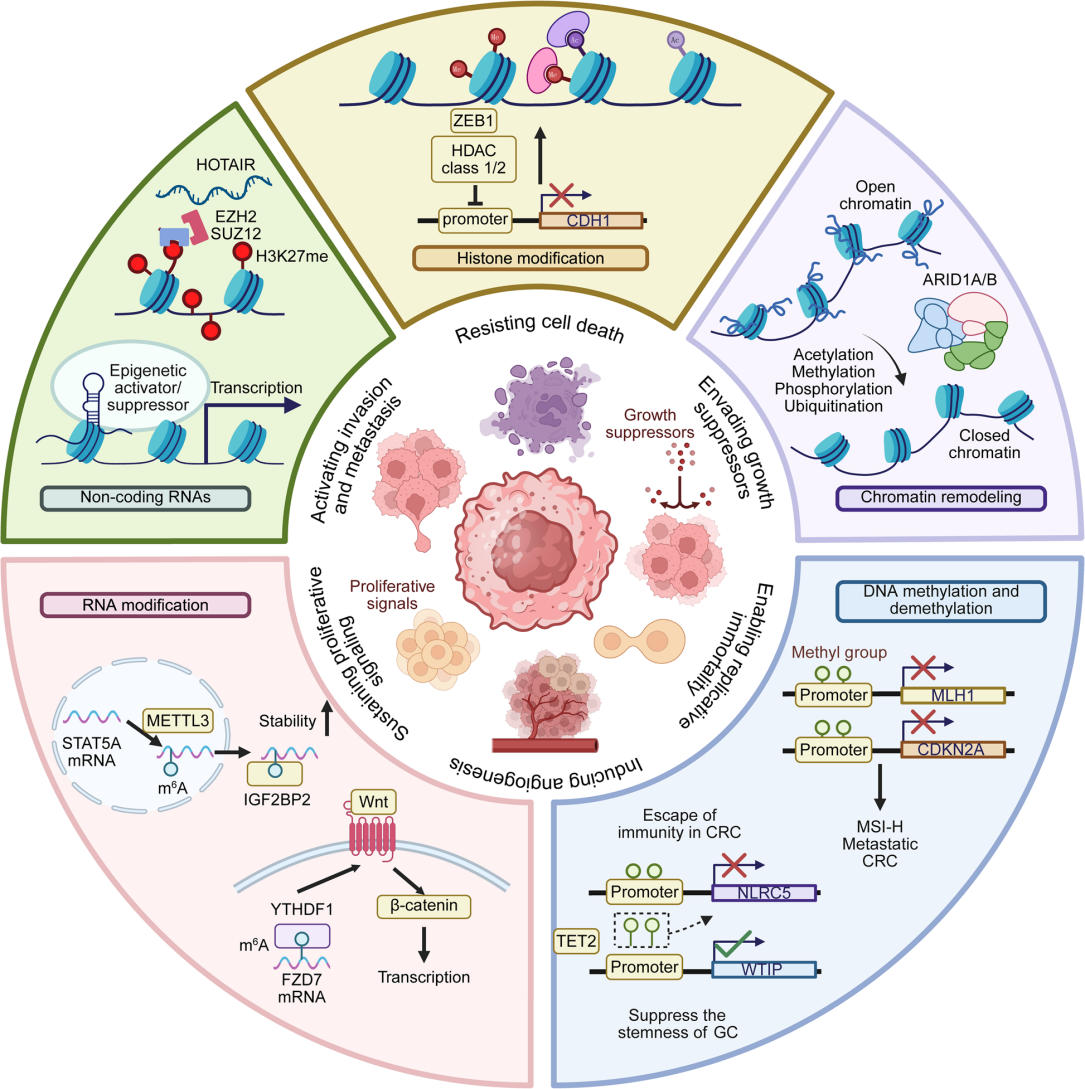
Epigenetic Regulation of Hallmark Capabilities in Gastrointestinal Tumor Progression. The epigenetic factors involved in the molecular mechanisms of GI tumors, categorized into five key aspects: non-coding RNAs, histone modification, chromatin remodeling, RNA modification, and DNA methylation/demethylation. Each section highlights specific processes and pathways contributing to tumor progression and resistance mechanisms. The inner circle represents the hallmark capabilities of cancer cells, including resisting cell death, activating invasion and metastasis, inducing angiogenesis, enabling replicative immortality, evading growth suppressors, and sustaining proliferative signals, all driven by the outer epigenetic mechanisms (figure was created with Biorender.com)

Epigenetic dysregulation is a fundamental mechanism driving GI tumorigenesis, acting in parallel with genetic events to influence chromatin state and gene expression [60]. Epigenetic regulation encompasses five key mechanisms: DNA modification, histone modification, RNA modification, chromatin remodeling, and non-coding RNA regulation (Fig. 2) [61].

#### Pancreatic cancer

Histone modifications also contribute significantly to GI tumorigenesis. Histone deacetylases (HDACs), particularly HDAC1 and HDAC2, are frequently upregulated in GI cancers. For example, in PC, ZEB1 recruits HDAC1/2 to the CDH1 promoter, causing histone H3/H4 deacetylation and transcriptional silencing of E-cadherin, which drives PC cell migration and proliferation [62]. In contrast, histone methyltransferases such as enhancer of zeste homolog 2 (EZH2), the catalytic subunit of the Polycomb Repressive Complex 2 (PRC2), are often overexpressed in PCs [63]. EZH2 catalyzes the addition of the repressive H3K27me3 mark, silencing tumor suppressor genes involved in cell cycle regulation and differentiation [64]. These histone modifications, whether through acetylation or methylation, create a permissive environment for tumor progression by altering chromatin accessibility and gene expression.

#### Colorectal cancer

Among these, DNA methylation plays a pivotal role, as seen in CRC, where the CpG Island Methylator Phenotype (CIMP) is characterized by widespread promoter hypermethylation. This leads to the silencing of tumor suppressor genes like *CDKN2A* and the MMR gene *MLH1*, resulting in MSI in sporadic tumors [65–67].Besides, CIMP is highly relevant to *BRAF* mutation in CRC [68].

A contradictory role of *NLRC5*, the master regulator of MHC class I genes, has been observed in different cancers, with low expression due to methylation linked to immune evasion in CRC while high expression is associated with GC progression [69–71]. This discrepancy may be attributed to cancer-specific immune escape strategies, genetic regulation, and TME differences [69].

In colorectal cancer, *ALKBH5* promotes tumor progression by stabilizing *RAB5A* mRNA, correlating with poor patient survival [72]. However, m6A modification can also act as a tumor suppressor in certain contexts. For example, in PC, *ALKBH5* removes m6A methylation to activate *PER1* expression, reactivating the ATM-CHK2-P53 signaling pathway to suppress tumor growth [73]. Furthermore, in CRC, *ALKBH5*-mediated m6A demethylation stabilizes the circular RNA circAFF2, enhancing radiosensitivity by inhibiting Cullin1 neddylation [74]. These findings highlight the dual role of m6A modification in GI cancers, functioning as both a promoter and suppressor of tumor progression depending on the context.

Other RNA modifications also play significant roles in GI cancers. Adenosine-to-inosine (A-to-I) RNA editing, mediated by enzymes like ADAR1, alters RNA base-pairing properties and is often dysregulated in malignancies. This process involves the deamination of adenosine, which converts it into inosine. As a result, the inosine base-pairs with cytosine in a way that mimics guanosine [75, 76].In CRC, elevated *ADAR1* expression increases the RNA editing of *AZIN1*, and this edited *AZIN1* functions as an oncogene by enhancing cancer cell stemness and driving metastasis [77, 78].

Additionally, OGT-mediated O-glycosylation promotes chemoresistance in CRC by stabilizing ADAR, which enhances its A-to-I editing activity on downstream targets like *PARP1*, thereby upregulating DNA repair capability [77]. Importantly, A-to-I editing has also been linked to tumor angiogenesis. In CRC, A-to-I RNA-edited *AZIN1* promotes angiogenesis by stabilizing c-Myc, which upregulates the angiogenic factor IL-8 [79]. This suggests that targeting IL-8 signaling could be a potential therapeutic strategy for cancers with this specific alteration. Beyond RNA editing, other modifications such as m5C methylation, pseudouridine modification, and ac4C acetylation contribute to GI cancer progression [80]. For example, the m5C methyltransferase NSUN2 initiates a positive feedback loop in CRC, modifying *ENO1* mRNA to boost lactate production, which in turn enhances NSUN2 expression and activity through lactylation, ultimately driving metabolic reprogramming and tumor progression [81, 82].

#### Gastric cancer

In terms of GC, G9a methylates RUNX3 at K129 and K171 under hypoxia, disrupting its interactions with cofactors, reducing acetylation and nucleocytoplasmic transport, thereby promoting cancer cell proliferation and tumor growth during early tumorigenesis [83]. These examples underscore the role of DNA methylation in silencing key genes that regulate tumor suppression and immune response. From the perspective of DNA demethylation, *TET2* suppresses GC chemoresistance by forming a transcriptional axis with *PATZ1* to upregulate *WTIP*, which interacts with PP2A to inhibit AKT T308 phosphorylation and activity, reducing stemness and chemoresistance, and its silencing suggests AKT inhibitors combined with chemotherapy as a potential treatment strategy for resistant GC [84].

RNA modifications, collectively referred to as epitranscriptomics, are another critical layer of epigenetic regulation in GI cancers. The most abundant internal modification on mRNA is N6-methyladenosine (m6A), which is dynamically regulated by “writer” enzymes like METTL3, “eraser” enzymes like FTO and ALKBH5, and “reader” proteins such as the YTHDF family [72]. In GC, the METTL3-IGF2BP2 axis promotes tumor progression by stabilizing STAT5A mRNA through m6A-dependent mechanisms, thereby repressing the tumor suppressor *KLF4* [85]. Similarly, YTHDF1 enhances the m6A-dependent translation of *FZD7*, hyperactivating the Wnt/β-catenin signaling pathway [86, 87].

Similarly, in GC, pseudouridine modification by PUS7 enhances the translation of tumor-suppressive protein *ALKBH3*, inhibiting tumor growth [88, 89]. Conversely, NAT10-mediated ac4C acetylation promotes GC progression by modifying *TNC* and *LDHA* mRNAs to enhance tumor growth and metabolic reprogramming [90].

Chromatin remodeling also plays a crucial role in GI tumorigenesis. *ARID1A*, a key component of the SWI/SNF chromatin remodeling complex, is frequently mutated in GC, particularly in MSI-H and EBV-associated subtypes, with mutations leading to protein truncation. Loss of ARID1A expression, correlated with its mutational status, is recognized as a poor prognostic factor in GC [91, 92].These mutations often result in loss-of-function phenotypes that impair tumor suppression. Also, ZEB1 interacts with the chromatin-remodeling protein BRG1 to repress E-cadherin expression, facilitating epithelial-to-mesenchymal transition and tumor invasion likely through chromatin remodeling [93]. Non-coding RNAs further add complexity to epigenetic regulation, interacting with RNA modifications and chromatin remodeling to influence tumor progression and therapeutic sensitivity. For example, circular RNAs like circAFF2, stabilized by m6A demethylation, enhance radiosensitivity by inhibiting Cullin1 neddylation [94]. They also play significant roles in GC as key epigenetic regulators, influencing gene expression through various mechanisms, such as HOTAIR (HOX transcript antisense RNA) [95, 96], SCHLAP1 (second chromosome locus associated with prostate-1) [97] and NEAT1(nuclear paraspeckle assembly transcript 1) [98].

#### Other GI cancer

Similarly, in esophageal squamous cell carcinoma, *ADAR1* overexpression enhances A-to-I editing of *AZIN1*, conferring a gain-of-function pro-invasive phenotype that leads to poor patient prognosis [99, 100]. In hepatocellular carcinoma (HCC), A-to-I RNA editing of *AZIN1* promotes tumorigenesis by stabilizing pro-proliferative proteins like ODC and cyclin D1, which bind more strongly to antizyme [101].

In summary, epigenetic dysregulation through DNA modification, histone modification, RNA modification, chromatin remodeling, and non-coding RNA regulation is central to GI tumorigenesis. These mechanisms are highly interconnected and context-dependent, acting as both oncogenic drivers and tumor suppressors. Understanding the dynamic interplay between these processes offers promising therapeutic opportunities for precision medicine in GI cancers.

### Key signaling pathways

#### Wnt/β-catenin pathway

The Wnt/β-catenin signaling pathway is a fundamental and highly conserved signal transduction cascade that governs cell fate decisions, proliferation, and tissue homeostasis throughout metazoan life. Its activity is centrally arbitrated by the cytoplasmic stability of the transcriptional co-activator β-catenin. In the pathway’s “off” state, a cytosolic destruction complex, scaffolded by Axin and the tumor suppressor APC, facilitates the sequential phosphorylation of β-catenin by Casein Kinase 1α (CK1α) and Glycogen Synthase Kinase 3β (GSK-3β). This phosphorylation marks β-catenin for ubiquitination by the β-TrCP E3 ligase complex and subsequent proteasomal degradation, thereby preventing its nuclear accumulation and keeping target gene transcription repressed by TCF/LEF transcription factors [102]. Conversely, pathway activation is initiated by the binding of a secreted Wnt glycoprotein to a cell-surface receptor complex composed of Frizzled (Fzd) and LRP5/6. This event triggers the recruitment of the Dishevelled (Dvl) protein, leading to the inhibition of the destruction complex and the consequent stabilization of β-catenin [103]. Accumulated β-catenin then translocates to the nucleus, where it displaces co-repressors and binds to TCF/LEF factors, recruiting transcriptional co-activators to drive the expression of target genes such as *c-Myc* and *Cyclin D1* [104]. Given its potent control over cellular growth and identity, it is unsurprising that aberrant constitutive activation of this pathway, which commonly refers to loss-of-function mutations in *APC* or gain-of-function mutations in *CTNNB1* (the gene encoding β-catenin), is a primary etiological driver in numerous human malignancies, most notably colorectal cancer [105].

CRC stands as the archetypal Wnt-driven malignancy, where dysregulation is typically an intrinsic, genetic, and initiating event in the well-established adenoma-carcinoma sequence. The primary mechanism is a “bottom-up” activation caused by the genetic inactivation of the β-catenin destruction complex, which leads to the formation of a stable, transcriptionally active β-catenin/TCF complex [105]. This is most commonly driven by loss-of-function mutations in the *APC* tumor suppressor gene, which are found in approximately 80–90% of cases [24]. In cases with wild-type *APC*, activating mutations in the *CTNNB1* gene serve as an alternative, mutually exclusive mechanism to make the protein resistant to degradation [106]. In this context, the mutations are sufficient to lock *Lgr5*-positive intestinal stem cells into a proliferative state, driving adenoma formation [107].

In stark contrast, Wnt/β-catenin activation in gastric and hepatocellular carcinomas is mechanistically more varied. In GC, activation is highly heterogeneous and less dominated by a single gene mutation [108]. A key mechanism is the epigenetic silencing of Wnt pathway antagonists, such as SFRPs, whose promoters are often hypermethylated [109]. Furthermore, the microenvironment plays a primary regulatory role, as chronic infection with *Helicobacter pylori (H. pylori)* allows the bacterial oncoprotein CagA to directly dysregulate and trigger β-catenin activation [110, 111]. HCC presents yet another distinct genetic profile. Here, the dysregulation is again primarily intrinsic, but the targets are different: activating mutations in *CTNNB1* are extremely common [112]. Large-scale genomic studies have confirmed that *CTNNB1* mutations (~ 30–40%) and inactivating mutations in *AXIN1* (~ 10%) are the dominant drivers, while *APC* mutations are notably rare [113, 114]. This specific mutational signature is often selected for in the context of chronic liver injury and inflammation, where a high-regeneration, pro-Wnt environment favors the growth of cells with these constitutive mutations.

A different scenario of “top-down” activation is observed in PDAC, where Wnt signaling is crucial but its activation is often a later event heavily influenced by the TME. Unlike in CRC or HCC, genomic analyses have shown that intrinsic mutations in core Wnt pathway components are very rare in PDAC, where the initiating driver is almost always a *KRAS* mutation [115]. Instead, the pathway is stimulated extrinsically by a dense desmoplastic stroma rich in cancer-associated fibroblasts (CAFs). These stromal cells are a major source of secreted Wnt ligands that act in a paracrine fashion to stimulate Wnt/β-catenin signaling in the cancer cells, supporting the proliferation of cancer stem cells and promoting invasion [116, 117].

Interaction with metabolic and kinase pathways: The LKB1/AMPK axis enhances Axin1 stability by phosphorylating the deubiquitinase USP10, promoting the formation of the β-catenin degradation complex while simultaneously inducing β-catenin phase separation to inhibit its nuclear translocation, thereby suppressing colorectal cancer growth [118]. Meanwhile, the PI3K/AKT pathway coordinates Wnt signaling through phospholipase D1 (PLD1), stabilizing β-catenin and promoting cancer stemness and chemoresistance [119]. When tumors exhibit high nuclear β-catenin and are treated with PI3K/AKT inhibitors, activated FOXO3a fails to induce apoptosis and instead cooperates with β-catenin to drive cell scattering and metastasis [120]. Upon activation by their ligands, receptor tyrosine kinases such as EGFR or c-MET phosphorylate and inhibit GSK-3β activity via the downstream PI3K/AKT signaling axis, thereby preventing β-catenin degradation and promoting its nuclear entry, which achieves a direct convergence of growth factor signaling and the Wnt pathway to potently drive tumor cell proliferation and survival [121]. The Hippo pathway effectors YAP/TAZ form complexes with β-catenin/TCF4 and TEAD to activate oncogenes (e.g., *MYC*), while VGLL4 competitively binds to TEAD to inhibit this complex formation, thereby blocking colon cancer metastasis [122, 123]. Meanwhile, the lncRNA CCAT5, when activated by β-catenin/TCF3, binds to STAT3 and prevents its dephosphorylation, driving peritoneal metastasis in gastric cancer [124]. CUB-domain-containing protein 1 potentiates the Wnt pathway in colorectal carcinoma by facilitating the nuclear accumulation of β-catenin and simultaneously driving E-cadherin into the nucleus [125].The TGF-β/Smad pathway competitively inhibits β-catenin/TCF transcriptional activity through Smad4, while Notch1 epigenetically silences Wnt target gene expression (e.g., by recruiting SET domain bifurcated 1 [SETDB1]), thereby exerting tumor-suppressive effects in colorectal cancer [126]. In digestive tumors driven by chronic inflammation, the NF-κB and Wnt pathways form a deadly positive feedback loop, where NF-κB activated by inflammatory signals synergizes with nuclear-accumulated β-catenin to convert a transient inflammatory response into a sustained, pro-cancerous signal for cell proliferation [127].

The ETS transcription factor ERG is an essential regulator of angiogenesis and vascular stability because it controls the Wnt/β-catenin pathway by promoting β-catenin stability through signals mediated by VE-cadherin and the Wnt receptor Frizzled-4 [128].

In the cancer stem cell niche of digestive tumors, the Hedgehog pathway engages in critical crosstalk with the Wnt pathway via its effector transcription factor GLI, either by directly upregulating Wnt ligand expression to activate Wnt signaling in neighboring cells in a paracrine manner, or by synergistically co-regulating a common set of downstream target genes with β-catenin in the nucleus, with both pathways working in concert to maintain the self-renewal capacity of cancer stem cells [129].

#### PI3K/AKT/mTOR pathway

The phosphatidylinositol 3-kinase (PI3K)/AKT/mammalian target of rapamycin (mTOR) signaling pathway is a critical intracellular cascade whose frequent dysregulation makes it a central driver of GI cancer progression [130]. The process is typically initiated when extracellular growth factors activate receptor tyrosine kinases (RTKs), which in turn recruit and activate PI3K. Activated PI3K then phosphorylates PIP2 to generate the second messenger PIP3, a process directly antagonized by the tumor suppressor PTEN [131]. This accumulation of PIP3 facilitates the recruitment and subsequent activation of the serine/threonine kinase AKT, which then orchestrates a potent pro-growth and pro-survival response. Furthermore, activated AKT stimulates mTOR Complex 1 (mTORC1), which was shown to directly phosphorylate key targets like the eukaryotic translation initiation factor 4E-binding protein 1 (4E-BP1) and p70 S6 kinase (S6K), unleashing protein synthesis required for cell growth [132]. In GI cancers, this cascade’s constitutive activation, commonly caused by activating mutations in the *PIK3CA* gene [133], loss of the PTEN tumor suppressor, or overload from upstream signals like KRAS, fuels tumor progression by enabling uncontrolled proliferation, evasion of apoptosis through the inhibition of proteins like BAD and FOXO [134], and the promotion of angiogenesis and metastasis. Large-scale genomic analyses of GI cancers have since confirmed that activating mutations in *PIK3CA* and loss-of-function alterations in *PTEN* are among the most common and clinically significant events, solidifying the pathway’s role as a core dependency in these tumors and a major focus for targeted therapy [24].

The activation of the PI3K signaling pathway varies significantly across different GI cancers, dictating distinct therapeutic vulnerabilities. In colorectal cancer, the pathway is most commonly activated by direct, therapeutically relevant mutations in the *PIK3CA* gene [24, 135]. In stark contrast, PC’s *PI3K* activation is almost always a secondary consequence of dominant upstream *KRAS* mutations, rendering direct PI3K inhibitors largely ineffective [136, 137]. Gastric and esophageal cancers exhibit more heterogeneous mechanisms; activation can occur through direct *PIK3CA* mutations (especially in EBV-positive gastric cancer and esophageal squamous cell carcinoma) or through the amplification of upstream receptors like *HER2* (in certain gastric and esophageal adenocarcinoma subtypes), making HER2 a key therapeutic target [4, 138]. Lastly, hepatocellular carcinoma pathway activation is often driven by the inflammatory TME and loss of the *PTEN* suppressor, responding best to multi-kinase inhibitors that block upstream signaling [114, 139].

#### MAPK/ERK signaling

In oncogenic contexts, the MAPK/ERK pathway transitions from a transiently regulated network, which normally responds to extracellular cues, into a constitutively active driver of malignant transformation [140, 141]. This dysregulation is most commonly caused by somatic, gain-of-function point mutations in key upstream nodes, particularly the *RAS* (e.g., *KRAS*, *NRAS*) and *RAF* (e.g., *BRAF*) families of proto-oncogenes, as comprehensively documented in large-scale cancer genome analyses [142, 143].

Specific mutations, such as those at codons 12, 13, and 61 in *KRAS*, impair the protein’s intrinsic GTPase activity, often by sterically hindering the binding of GTPase-activating proteins (GAPs), which are necessary to turn the signal off [144]. This impairment locks RAS in a perpetually GTP-bound, active state [145]. Similarly, the prevalent V600E mutation in *BRAF* introduces a negatively charged glutamic acid into the activation loop, which electrostatically mimics phosphorylation [146]. This disrupts the inactive conformation of the kinase domain, rendering it constitutively active and independent of upstream RAS signaling [147].

This ligand-independent activation results in persistent, high-level phosphorylation of MEK and ERK, thereby uncoupling the entire cascade from its physiological regulation [147]. The resulting sustained nuclear ERK activity is a potent oncogenic force, directly altering the expression of a vast network of over 700 target genes that orchestrate the hallmarks of cancer [148]. For instance, activated ERK phosphorylates and activates transcription factors that upregulate cyclins (e.g., Cyclin D1) to drive relentless G1/S phase progression [149]. It also confers resistance to apoptosis by directly phosphorylating and inactivating pro-apoptotic BCL-2 family members like BIM and BAD [150, 151], and it promotes metabolic reprogramming, including the Warburg effect, to fuel relentless proliferation [152].

In the landscape of GI cancers, this constitutive signaling is not merely an aberration but a core oncogene addiction, a state where cancer cells are exquisitely dependent on this single activated pathway for their viability and proliferation [153, 154]. This profound dependency is precisely what establishes the pathway as a validated and critical target for therapeutic intervention with specific inhibitors [155].

The current research landscape for the MAPK/ERK pathway in GI cancers has evolved from simply identifying its role to developing sophisticated strategies to counteract its effects. A primary focus is on overcoming therapeutic resistance, particularly in colorectal cancer, where the initial failure of single-agent BRAF inhibitors led to the discovery that feedback activation of EGFR reactivates the pathway [156]. This crucial insight established the current standard of care about combining BRAF and EGFR inhibitors and has spurred further investigation into triplet therapies to preemptively block new resistance mechanisms [157]. In parallel, the once “undruggable” *KRAS*, a key driver in PC, has become a major therapeutic target with the advent of direct KRAS G12C inhibitors [158]. However, as resistance through pathway reactivation remains a significant hurdle, current research is heavily focused on creating more durable responses by combining these new inhibitors with agents targeting other nodes in the pathway, such as SHP2 [159]. Beyond direct inhibition, researchers are exploring innovative strategies like synthetic lethality, exemplified by the development of WRN helicase inhibitors, which are specifically toxic to cancer cells with MAPK pathway mutations [160]. Furthermore, the field is increasingly recognizing the pathway’s critical role in shaping the TME; studies now show that MAPK signaling fosters an immunosuppressive climate, and inhibiting it can enhance the efficacy of immunotherapies by making cancer cells more vulnerable to an anti-tumor immune attack [161].

#### p53 Axis

The *TP53* gene encodes a transcription factor that functions as a central node in a network integrating cellular stress signals to control cell fate. Under normal homeostasis, wild-type p53 activity is exquisitely regulated, primarily through its interaction with the E3 ubiquitin ligase MDM2, which targets p53 for proteasomal degradation [162]. However, this regulation is far more complex than a simple two-protein loop, involving a host of deubiquitinating enzymes (DUBs) that counteract MDM2 [163]. For instance, recent work has shown that the DUB OTUD5 can directly stabilize p53 following DNA damage, and its loss impairs the p53-dependent DNA damage response, highlighting the dynamic and contested nature of p53 stability [164]. Upon genotoxic insult, this homeostatic balance is deliberately broken by stress-activated kinases, which phosphorylate p53 to disrupt MDM2 binding and recruit co-activators that acetylate p53, a modification essential for activating its sequence-specific DNA binding and transcriptional power [165, 166].

Consequently, the inactivation of this crucial signaling axis is a cornerstone of GI tumorigenesis. Large-scale pan-cancer analyses of whole genomes have reinforced that *TP53* is the most frequently mutated gene across human cancers, with a particularly high prevalence in GI malignancies [67]. The timing of this event is a critical determinant of tumor evolution. In colorectal cancer, *TP53* mutation remains a key late event driving malignant progression. In contrast, detailed multi-region sequencing of pancreatic tumors has provided definitive evidence that *TP53* inactivation is an early, trunk event in pancreatic ductal adenocarcinoma, essential for clonal expansion [167]. Likewise, genomic analyses tracking the evolution of esophageal adenocarcinoma from its precursor lesion, Barrett’s esophagus, have confirmed that *TP53* mutation or loss of heterozygosity is the most significant driver of progression to invasive cancer [168].

Furthermore, the functional consequences of *TP53* alterations extend far beyond simple loss-of-function, primarily because the mutational landscape is dominated by missense mutations that yield a stable, full-length protein. These mutant proteins not only exert a dominant-negative effect on any remaining wild-type p53 but, more importantly, acquire entirely new, oncogenic capabilities known as gain-of-function activities. Recent mechanistic studies have revealed precisely how these GOF activities operate. For example, specific p53 mutants have been shown to rewire chromatin landscapes, creating novel enhancer regions that drive the expression of pro-invasive genes and confer chemoresistance in gastric cancer [169].

This detailed molecular understanding has therefore paved the way for targeted therapeutic strategies. For tumors harboring *TP53* missense mutations, agents designed to restore wild-type function are in active clinical development. APR-246 (eprenetapopt), a covalent modifier of mutant p53, has shown clinical activity in hematologic malignancies and is under investigation in solid tumors [170]. Conversely, for tumors that retain wild-type *TP53* but inactivate the pathway via *MDM2* amplification, a different strategy is required. Here, potent inhibitors of the MDM2-p53 interaction, such as navtemadlin (AMG-232), are being tested in clinical trials and have demonstrated molecular and clinical responses in patients with *TP53* wild-type solid tumors, validating the approach of liberating p53 from its negative regulator [171]. Thus, the specific molecular state of the *TP53* gene itself dictates the rational therapeutic approach for restoring this critical guardian pathway.

#### Other signaling pathways

In the complex landscape of GI carcinogenesis, several interconnected signaling networks orchestrate tumor progression, particularly by governing the crucial interplay between cancer cells and their microenvironment. Beyond the foundational pathways, a deeper understanding of these networks is revealing critical vulnerabilities that can be targeted for therapy.

At the core of tumor growth are pathways that directly regulate cell proliferation and fate. The Hippo pathway, a key tumor suppressor network that controls organ size, is frequently inactivated in GI cancers. This inactivation unleashes its downstream effectors, YAP and TAZ, which drive uncontrolled cell growth. Crucially, research has shown this is not just an internal process. Canonical Hippo kinases (MST1/2–LATS1/2) restrain YAP/TAZ nuclear co‑activators; their functional attenuation in GI tumors arises via genomic lesions [172, 173] or epigenetic silencing, or, more prominently, by microenvironmental forces. Calvo et al. demonstrated that fibroblast activation and extracellular matrix (ECM) stiffening form a mechanotransduction loop that locks YAP/TAZ in an active state, sustaining cancer-associated fibroblast (CAF) phenotypes and invasive niches YAP/TAZ integrate signals from integrins, focal adhesion kinase (FAK), Src, Rho GTPases, and Wnt destruction complex components to coordinate proliferation with mechanical inputs [174, 175]. In PDAC, *YAP1* activation can substitute for oncogenic *KRAS*, furnishing a bypass route of lineage‑survival signaling and adaptive resistance after KRAS/MAPK suppression [176]. YAP/TAZ drive metabolic rewiring, redox stress buffering, and nucleotide biosynthesis, helping tumor cells survive hypovascular, nutrient‑poor desmoplastic regions [177]. They also upregulate immune checkpoint ligands and cytokines that recruit suppressive myeloid cells, while influencing cancer stem-cell plasticity and minimal residual disease after chemotherapy [178].

Research across several GI cancers highlights the Notch signaling pathway as a critical driver of tumor progression, primarily by maintaining cancer stem cells and enabling therapy resistance. For instance, Ridgway, J. et al. demonstrated in multiple tumor models that targeting the Notch ligand DLL4 effectively inhibits tumor growth by suppressing angiogenesis [179]. Once a tumor is established, cancer cells actively manipulate their surroundings using other pathways. The Hedgehog (Hh) pathway is a prime example of this, functioning through a “paracrine” mechanism where tumor cells secrete Hh ligands to activate adjacent stromal fibroblasts. Seminal work by Steele et al. in PC models revealed that this activated stroma creates a dense, protective barrier that both physically constrains the tumor and impedes the delivery of therapeutic drugs, highlighting the immense challenge of targeting this tumor-stroma crosstalk [180].

Ultimately, the TGF-β pathway acts as a master regulator that integrates these diverse processes. While it functions as a tumor suppressor in early-stage disease, it undergoes a functional switch in advanced GI cancers to become a powerful driver of malignancy. Its pro-tumorigenic role is multifaceted. It can induce cancer cells to undergo an EMT, a key step in metastasis, and it profoundly shapes the TME. A study by Calon et al. uncovered a specific mechanism for this, showing that TGF-β-activated fibroblasts secrete proteins that prepare distant organs, like the liver, to receive and host metastatic cells [181]. In a “quadruple-mutant” model of colorectal cancer, TGF-β signaling acts as a primary defense mechanism that shields the tumor by both physically excluding cancer-killing T-cells and deactivating any that manage to get close [182]. Through the analysis of a large number of clinical samples, it was discovered that TGF-β signaling is a primary reason for the exclusion of T-cells from the tumor core, which in turn directly leads to the ineffectiveness of PD-L1 inhibitors [183]. In PC models, it was shown that TGF-β not only drives dense fibrosis, which obstructs chemotherapy drug delivery, but also inhibits the anti-tumor immune response through various mechanisms [184].

### TME

TME of GI malignancies is fundamentally distinct from that of other solid tumors, such as melanoma or lung carcinoma [185, 186]. The singular nature of the GI TME is principally governed by the confluence of two core elements: the direct immunomodulatory activity of the gut microbiota and the inherent backdrop of mucosal immune tolerance.

#### Direct immunomodulatory role of the gut microbiota

Unlike TMEs within sterile or low-microbial-burden tissues, the GI TME is in direct and continuous interface with the complex microbial ecosystem of the gut. These resident microorganisms and their metabolic byproducts are not passive constituents but rather integral and active modulators of the local tumor milieu. A salient example is the pathogenic activity of *Fusobacterium nucleatum* [187]. This bacterium has been demonstrated to promote immune evasion through at least two distinct mechanisms: its Fap2 protein can directly engage the Gal-GalNAc receptor on tumor cells, and it can also interact with the T-cell immunoreceptor with Ig and ITIM domains (TIGIT) on natural killer (NK) cells and T cells [188–190]. Both interactions culminate in the suppression of cytotoxic anti-tumor responses, thereby fostering an immunosuppressive microenvironment [191, 192]. Such direct, microbially-driven modulation of anti-tumor immunity represents a defining characteristic of GI carcinogenesis that is largely absent in other oncological contexts [188].

The GI TME develops against a pre-existing physiological backdrop of profound immune tolerance. The primary mandate of the mucosal immune system is to maintain a state of non-reactivity towards innocuous dietary antigens and the commensal microbiota, a process essential for preventing chronic inflammation [193]. This inherent, homeostatic immunosuppressive bias can be systematically exploited during oncogenesis. The GI mucosa is naturally characterized by a high prevalence of regulatory T cells (Tregs) and other cellular populations that secrete immunosuppressive cytokines, notably Transforming Growth Factor-beta (TGF-β) and Interleukin-10 (IL-10) [194, 195]. Malignant cells can co-opt and potentiate these pre-established regulatory pathways to facilitate immune escape, obviating the need to construct an immunosuppressive network de novo [196]. In colorectal carcinoma, for instance, tumor cells frequently upregulate TGF-β signaling, which promotes the differentiation and expansion of Tregs while concurrently inhibiting the effector functions of CD8^+^ T cells [197, 198]. This represents a malignant appropriation of a fundamental mechanism of mucosal homeostasis.

Although HCC and PDAC are not anatomically intraluminal tumors, they are nevertheless subjected to precise molecular regulation by the gut microbiota and its derived signals via the gut-liver and gut-pancreas axes [199]. In HCC, the core mechanism lies in the direct reprogramming of hepatic immune cells by microbe-associated molecular patterns (MAMPs): translocated bacterial lipopolysaccharide (LPS) activates intrahepatic Kupffer cells via the TLR4-MyD88-NF-κB signaling axis, triggering massive secretion of IL-6 and TNF-α [200, 201]. These cytokines not only directly promote hepatocyte proliferation and induce DNA damage but also lead to sustained activation of the STAT3 pathway, collectively shaping a pro-tumor inflammatory TME [202]. In PDAC, microbial influence is more direct, as well as TLR4-MyD88-NF-κB signaling axis [202]. Specific pathogens such as Fusobacterium nucleatum can translocate to the pancreas, where its Fap2 protein not only binds to Gal-GalNAc on PC cells to enhance their proliferative and invasive capabilities but also directly engages the TIGIT receptor on tumor-infiltrating NK cells and CD8^+^ T cells, suppressing their cytotoxicity and thereby establishing an immune-exempt microenvironment [189, 203]. Furthermore, gut microbiota-derived secondary bile acids, such as deoxycholic acid (DCA), primarily exert cytotoxic effects by inducing oxidative stress and DNA damage, driving hepatic stellate cell activation in HCC and exacerbating the fibrotic stromal response in PDAC [204].

#### Tripartite metabolic competition and crosstalk among the host, microbiota, and tumor

The metabolic landscape of the GI TME is uniquely complex, defined by a tripartite interplay involving intense competition for nutrients and intricate metabolic exchanges between host cells, tumor cells, and the resident gut microbiota [205]. This three-way interaction establishes a distinct metabolic ecosystem not observed in other tumor types.

A paradigmatic example of this complexity is the role of short-chain fatty acids (SCFAs), particularly butyrate, which are produced through microbial fermentation of dietary fiber [206, 207]. On one hand, it serves as the primary energy source for healthy colonocytes and functions as an endogenous HDAC inhibitor due to the Warburg effect, which can induce apoptosis and suppress proliferation in colorectal cancer cells [208, 209]. On the other hand, within the established TME, butyrate can exert potent immunomodulatory effects that may paradoxically favor tumor progression [210]. For instance, it has been shown to enhance the differentiation and suppressive function of Tregs and modulate macrophage activity, thereby contributing to an immunosuppressive milieu [211].

This direct intervention by metabolites produced by a third party, the microbiota, which can dually regulate the interaction between tumor and immune cells, represents a core and defining feature of the metabolic architecture of the GI TME.

#### TME heterogeneity in major GI cancers

Although TME has some common components and functional principles, it is not a “one-size-fits-all” entity. Instead, TME’s characteristics are deeply “shaped” or “flavored” by the unique biological and pathophysiological context of the organ in which it resides. This organ specificity determines the dominant cell types in the TME, key signaling pathways, and ultimately the therapeutic targets.

In gastric cancer, the formation and evolution of the TME are intricately linked to chronic *Helicobacter pylori* infection, which stands as the most potent single risk factor for this malignancy [212, 213]. *H. pylori* and its secreted virulence factors, such as cytotoxin-associated gene A protein (CagA) and vacuolating cytotoxin A (VacA), trigger a persistent chronic inflammatory response like epithelial differentiation in the gastric mucosa [214–216]. *H. pylori* infection reshapes the gastric microenvironment through several mechanisms:

First, recruitment and polarization of immune cells. It induces significant infiltration of neutrophils and macrophages. The reactive oxygen species (ROS) and reactive nitrogen species (RNS) released by these cells can directly cause DNA damage in gastric epithelial cells [217, 218]. Concurrently, *H. pylori* can induce the polarization of macrophages towards an M2 phenotype and promote the recruitment and activation of myeloid-derived suppressor cells (MDSCs), thereby establishing an immunosuppressive environment. The strategic manipulation of endogenous reactive oxygen species to attain bactericidal levels offers a promising and innovative therapeutic approach for effectively combating *H. pylori* infections. Second, activation of stromal cells. Infection can activate normal gastric fibroblasts into CAFs with pro-tumorigenic properties. These CAFs support tumor growth by secreting growth factors and remodeling the extracellular matrix [219, 220]. Third, promotion of immune evasion, *H. pylori* can impair the antigen-presenting function of dendritic cells and upregulate the expression of immune checkpoint molecules, such as PD-L1, on the surface of tumor cells and immune cells, thereby assisting tumor cells in evading immune surveillance [221, 222].

The unique feature of the TME in CRC lies in its close association with the gut microbiome. The trillions of microorganisms within the gut form a dynamic component of the TME, profoundly influencing the immune “temperature” of CRC. Clinically, CRC can be broadly categorized into “hot tumors” and “cold tumors” based on the degree of immune infiltration.

“Hot tumors” generally refer to tumors characterized by significant infiltration of immune cells, particularly CD8^+^ T cells, and are highly responsive to immune checkpoint inhibitor (ICI) therapy. Such tumors frequently exhibit dMMR or MSI-H, leading to the generation of numerous neoantigens that elicit a robust immune response [223]. Research indicates that in the TME of dMMR/MSI-H CRC, the gut microbiota displays greater diversity and abundance, being enriched with beneficial bacteria like *Akkermansia* and *Bifidobacterium* [224–226]. These microbial communities and their metabolites (e.g., short-chain fatty acids) can enhance immune cell activity and infiltration, thereby sustaining the “hot” state of the TME [227].

“Cold tumors,” in contrast, are characterized by sparse immune cell infiltration and are generally unresponsive to ICI therapy [228]. The majority of CRC are classified as proficient mismatch repair (pMMR) or microsatellite stable (MSS) subtypes, which are typically considered “cold tumors” [229]. The TME of these cancers is often associated with low microbial diversity and may harbor immunosuppressive bacterial species, such as Fusobacterium nucleatum [230]. These bacteria contribute to the establishment of an immunosuppressive “cold” environment by promoting the polarization of tumor-associated macrophages (TAMs) toward the M2 phenotype, recruiting myeloid-derived suppressor cells (MDSCs) and Tregs, and thereby suppressing anti-tumor immune responses [231, 232].

The TME of PDAC is characterized by its highly dense and fibrotic stroma (desmoplasia), which represents its most distinctive pathological feature and a critical factor contributing to the extreme difficulty of treatment [233, 234]. In PDAC, more than 70%–90% of the tumor volume is composed of stromal components rather than tumor cells [235, 236]. This stromal barrier, predominantly orchestrated by CAFs, facilitates the malignant behavior of PDAC through multiple mechanisms.

CAFs are the primary producers and maintainers of the dense stroma. They not only secrete ECM components but also release a plethora of growth factors, cytokines, and chemokines, thereby establishing a strongly pro-tumorigenic and immunosuppressive signaling network. For example, CAF-derived CXCL12 actively repels effector T cells from infiltrating the tumor, creating an “immune-exclusionary” microenvironment that further exacerbates immune evasion [237, 238].

HCC predominantly arises in the context of chronic liver diseases such as viral hepatitis, alcoholic liver disease, and non-alcoholic steatohepatitis, which lead to fibrosis and cirrhosis [239]. The liver’s inherent immune tolerance is amplified within the HCC TME, resulting in a profoundly immunosuppressive and fibrotic setting. Chronic liver injury activates hepatic stellate cells (HSCs), which differentiate into myofibroblasts and secrete ECM, driving fibrosis [240, 241]. The immune-tolerant environment of the liver is further exploited by HCC through the amplification of immunosuppressive cells such as Kupffer cells, cells Tregs, and myeloid-derived suppressor cells (MDSCs) [242]. Kupffer cells, liver-resident macrophages, express PD-L1 and secrete cytokines like IL-10 and TGF-β to suppress cytotoxic T-cell activity, while Tregs and MDSCs inhibit effector T-cell responses via cytokine signaling and metabolic disruption. Additionally, liver-specific immune cells such as Kupffer cells and liver sinusoidal endothelial cells (LSECs) play specialized roles in tumor-associated inflammation and immune tolerance, contributing to immune suppression and tumor progression. This pre-existing fibrotic and immune-tolerant organ environment explains the limited efficacy of immune checkpoint inhibitors in HCC and highlights the need for combination strategies targeting both immune suppression and stromal remodeling to improve therapeutic outcomes.

Genetic factors, signaling pathways, and the tumor microenvironment collectively exert synergistic regulatory effects on the initiation and progression of gastrointestinal cancers. Specifically, genetic mutations provide the initial driving forces, signaling pathways mediate a series of malignant phenotypes, and the tumor microenvironment primarily shapes immunosuppression and fosters therapy resistance. Under this synergistic regulation, effective therapeutic strategies often require a shift from single-target interventions to multi-layered approaches that integrate genomic characteristics, pathway crosstalk, and microenvironment remodeling.

## Diagnostic biomarkers and techniques

### Biomarkers in early detection

A common trend in recent studies is the identification of non-invasive or minimally invasive biomarkers for the early diagnosis of major GI cancers, such as colorectal and gastric cancers [243–245]. These investigations collectively reveal a clear paradigm shift, with research focus transitioning from traditional tissue biopsies to biomarkers derived from blood (serum/plasma) and fecal samples, as these sample types are more accessible and suitable for large-scale screening.

In terms of specific research directions, various types of biomarkers are being explored. Compared to the clinical biomarkers emphasized in early studies by Srivastava et al. (2001), recent research has increasingly concentrated on molecular biomarkers [246]. For instance, studies by Huang et al. (2010) and Zhu et al. (2014) demonstrated that microRNAs (miRNAs) present in plasma hold significant promise as novel biomarkers for the early detection of colorectal and gastric cancers [247, 248]. This body of work highlights a growing interest in non-coding RNAs, including miRNAs and piRNAs, which have emerged as highly promising candidates due to their stability in the circulatory system [247–249].

In contrast to the focus on circulating nucleic acids, other studies have emphasized epigenetic biomarkers. For example, Chen and Fang [250] (2015) explored the application of epigenetic modifications, such as DNA methylation, in the early diagnosis of gastric and colorectal cancers. Bernal et al. investigated the tumor suppressor gene Reprimo, identifying hypermethylation in its promoter region in gastric cancer tissues [251]. Importantly, these methylated DNA fragments are shed into the bloodstream and can be detected in plasma samples. Furthermore, Solé et al. (2014) presented a prototypical workflow for proteomics-based screening in colorectal cancer, underscoring the diversity of approaches employed in biomarker discovery [245]. Collectively, these studies demonstrate that researchers are investigating biomarkers across multiple levels, including genetic, epigenetic, and transcriptomic dimensions.

Despite the significant focus on the discovery and validation of novel biomarkers, there remains a notable gap in the integration of these biomarkers into clinical practice, as well as in conducting large-scale, prospective clinical validation studies [245, 248]. Additionally, while research on colorectal cancer and gastric cancer is relatively extensive, studies on early detection biomarkers for other GI cancers, such as esophageal and PCs, remain limited. This represents a significant gap in the current research landscape [243, 244, 251].

Current research on early cancer detection focuses on integrating multi-omics data with machine learning to enhance sensitivity and specificity. By parallel analysis of circulating tumor DNA genomic mutations, epigenomic methylation patterns, and proteomic data, combined with machine learning models for classification, this strategy has demonstrated clinical feasibility in multi-cancer detection and tumor tissue-of-origin (TOO) tracing [252]. Among ctDNA analyses, methylation patterns, as stable epigenetic markers, provide superior information for TOO tracing compared to genomic mutations, as evidenced by PanSeer’s methylation profiling achieving 80.6% sensitivity for early gastric cancer detection (Taizhou Longitudinal Study) [253]. Additionally, fragmentomics analyzes ctDNA’s physical attributes, such as fragment length distribution and terminal sequence patterns, enabling cancer detection without relying on driver mutations [254]. Another approach involves targeting tumor-derived extracellular vesicles (EVs) using tools like nucleic acid aptamers to enrich signals [255, 256]. Expanding beyond tumor-centric perspectives, research has integrated host metabolic and microbiome responses, combining plasma metabolomics, proteomics, and stool metagenomics to develop high-performance screening models, such as a panel of nine plasma metabolites achieving an AUC of 0.94 for early PC detection [257, 258]. These strategies collectively offer multi-dimensional solutions for advancing early cancer screening.

### Advanced imaging techniques

In recent years, rapid advancements in imaging technologies have significantly enhanced the early diagnosis, precise staging, and treatment evaluation of GI tumors. Commonly used clinical screening methods include computed tomography (CT) and ultrasound, with abdominal ultrasound being a preferred option due to its non-invasive nature, cost-effectiveness, and widespread availability. CT, with its high spatial resolution and multiphase enhancement capabilities, is extensively utilized for staging and diagnosis of diseases like liver and PCs [259, 260].

Emerging technologies, such as magnetic resonance imaging (MRI) and its functional imaging techniques, including diffusion-weighted imaging (DWI), dynamic contrast-enhanced MRI (DCE-MRI), and magnetic resonance spectroscopy (MRS), offer detailed insights into microstructural, hemodynamic, and metabolic features, significantly improving diagnostic accuracy. However, their high costs restrict their use to complex or challenging cases.

Positron emission tomography (PET), using tracers like 18F-fluorode-oxyglucose (18F-FDG), excels in detecting distant metastases by evaluating tumor metabolic activity, though its high cost and radiation exposure limit widespread adoption [261, 262]. Contrast-enhanced ultrasound (CEUS) effectively visualizes tumor perfusion characteristics, particularly for liver cancer diagnosis and treatment monitoring, but remains less sensitive to deep lesions [263].

Innovative techniques such as confocal laser endomicroscopy (CLE) and capsule endoscopy have entered clinical practice. CLE provides real-time cellular-level imaging, enabling direct visualization of tumor microstructures [264]. Capsule endoscopy, a non-invasive method, significantly improves screening for small intestine and GI tumors, including esophageal cancer. However, its inability to perform biopsies limits diagnostic capacity [265]. Despite this, it holds great potential for reaching areas inaccessible to traditional endoscopy, such as the duodenum, jejunum, and ileum.

Artificial intelligence (AI) has further revolutionized imaging diagnostics by enhancing efficiency and precision [266]. Deep learning algorithms integrate multimodal data from MRI, CT, and PET to enable tumor boundary detection, volumetric quantification, and treatment outcome prediction, offering valuable support in clinical decision-making [267–270].

Overall, conventional screening techniques like CT and ultrasound remain suitable for large-scale applications due to their affordability and accessibility. In contrast, advanced technologies such as MRI, CLE, and AI-assisted diagnostics are better suited for precise evaluations and complex cases [259, 271]. By integrating anatomical, functional, metabolic, and microstructural information, these imaging modalities provide critical insights for early diagnosis and personalized treatment planning. With ongoing technological progress, their clinical potential continues to expand.

## Current targeted therapies for GI cancer

### Monoclonal antibodies and small molecular drugs

Monoclonal antibodies and small molecule inhibitors are widely applied as essential strategies for targeted therapy in GI cancers. Monoclonal antibodies target cell surface receptors (e.g., EGFR, HER2) or components of the TME (e.g., VEGF), blocking pro-proliferative signaling or inhibiting angiogenesis. Small molecule inhibitors, on the other hand, penetrate cell membranes and act on intracellular kinases or signaling molecules, disrupting downstream oncogenic pathways. With deeper understanding of tumor molecular mechanisms and advancements in biomarker detection technologies, the application of targeted drugs is gradually moving toward personalized treatment, significantly improving clinical outcomes for specific subtypes of patients. Meanwhile, innovative drug development targeting emerging targets continues to drive progress in therapeutic approaches.

#### Monoclonal antibodies

Currently, several monoclonal antibodies have been approved by the FDA and are widely used in clinical practice, primarily targeting EGFR, VEGF, and HER2 pathways. These drugs have demonstrated significant efficacy in advanced GI cancers.

The EGFR signaling pathway is a key driver of proliferation, angiogenesis, and metastasis in many GI tumors. For example, pivotal clinical trials such as CRYSTAL and PRIME have shown that cetuximab and panitumumab significantly improve progression-free survival (PFS) and overall survival (OS) in RAS wild-type metastatic colorectal cancer (mCRC) patients, but are ineffective in *RAS*-mutant tumors [272–278]. In addition to EGFR, antibodies targeting angiogenesis have also been approved for treatment, such as bevacizumab and ramucirumab, which inhibit VEGF and VEGFR-2, respectively, significantly delaying tumor progression. Bevacizumab combined with chemotherapy has been shown in clinical trials to extend PFS by nearly six months, while ramucirumab extended OS by nearly two months (9.6 months vs. 7.4 months) in phase III trials for advanced gastric cancer [279–281]. Furthermore, immunotherapy combined with anti-angiogenesis drugs has demonstrated exceptional efficacy as IMbrave150 trial results showed that atezolizumab combined with bevacizumab extended OS and PFS in advanced HCC compared to sorafenib monotherapy [282]. For HER2-positive gastric cancer, trastuzumab has shown significant efficacy when combined with chemotherapy in the ToGA trial (NCT01041404), becoming a classic example of HER2-targeted therapy [3, 283].

Several monoclonal antibodies in clinical trials have also demonstrated promising therapeutic potential. Zolbetuximab targets Claudin-18.2 and significantly improved OS and PFS in Claudin-18.2-positive gastric cancer patients in the SPOTLIGHT trial (10.61 vs. 8.67 months) [284]. Another trial explored its application in gastric or gastroesophageal junction adenocarcinoma, providing data for expanding its treatment scope [285]. Necitumumab, targeting EGFR, has shown early efficacy in mCRC treatment when combined with chemotherapy [286]. Bemarituzumab, targeting the FGFR2b receptor, demonstrated efficacy in combination with mFOLFOX6 for metastatic gastric/gastroesophageal junction adenocarcinoma in the FIGHT trial (median PFS 9.5 vs. 7.4 months), with phase III clinical trials currently underway [287].

Building upon the development of single-epitope antibodies, biparatopic antibodies are emerging as a promising novel strategy. HER2-targeting biparatopic antibodies achieve dual blockade of the HER2 signaling pathway by engaging distinct structural domains of HER2. Regarding HCC, a novel anti-HER2 biparatopic tetravalent antibody exhibits enhanced antitumor efficacy through multiple mechanisms, including inducing HER2 clustering, internalization, and degradation [288–292]. In a clinical trial targeting both EGFR and MET, amivantamab achieved a disease control rate of 26.1% in patients with gastric cancer and gastroesophageal junction cancer [293]. Of particular note, Zanidatamab was formally granted Orphan Drug Designation by the FDA in 2024 for the treatment of biliary tract cancer and GC, which efficacy in gastroesophageal adenocarcinoma has been demonstrated in various phase I, II and III clinical studies (NCT03929666) [294–297]. According to findings from the KC-WISE/KN026-001 trial (NCT05427383), it demonstrates superior PFS and OS outcomes both in GC and CRC [298]. Moreover, in 2024, the HER2/HER3 bispecific antibody Zenocutuzumab was approved for treating NRG1^+^ PC [299]. These research findings provide important references for developing therapeutic strategies based on biparatopic antibodies.

Antibody-drug conjugates (ADCs) combine monoclonal antibodies with highly potent cytotoxic payloads via chemical linkers. This design leverages the targeting specificity and long half-life of antibodies while utilizing the high cytotoxicity of anti-tumor agents that cannot be used alone due to excessive toxicity [300]. FDA-approved ADCs include trastuzumab deruxtecan (Enhertu), targeting HER2 for HER2-positive gastric and gastroesophageal junction adenocarcinoma; its DESTINY-Gastric01 trial showed significant efficacy [301, 302]. Furthermore, the clinical trial that led to the approval of the EGFR-targeting ADC in nasopharyngeal carcinoma established its therapeutic utility in colorectal cancer [303]. Besides, HER2-targeting ADCs under investigation include RC48-ADC, A166, and ZW49, all showing good safety and efficacy [304, 305]. In recent years, researches on TROP-2 and CLDN18.2 have been boosted, demonstrating their manageable safety profile [306–310]. ADCs in clinical trials include sacituzumab govitecan, targeting TROP-2 for TROP-2-expressing gastric and colorectal cancers [311, 312]. Additionally, B7-H3-targeting ADC DS-7300 and novel TROP-2-targeting ADCs are being evaluated in phase I trials. Additionally, novel emerging ADC targets including HER3, Nectin-4, and GCC are currently under exploration. The innovation of HER3-targeted ADCs lies in their ability to disrupt HER2/HER3 heterodimerization and downstream signaling pathways, providing new strategies to overcome HER2-targeted therapy resistance [313]. Nectin-4, as a cell adhesion molecule highly expressed in gastric cancer, offers a new direction for cross-tumor translational therapy in gastric cancer [314]. The GCC target has garnered significant attention due to its highly specific expression in gastrointestinal mucosa, with its ADC exploration primarily facing challenges in balancing efficacy and toxicity [315, 316]. Moreover, GPC3 in HCC (MRG006A) (NCT07093970), CEACAM5 in mCRC (Precem-TcT) [317] and TF in PC (XNW28012) (NCT06799637) are all in clinical trial stages, demonstrating tremendous potential. Furthermore, based on the previously mentioned bispecific antibodies, researchers have conceived adopting similar approaches to conjugate them with drugs to form ADCs, such as Zanidatamab zovodotin (ZW49), which combines an anti-HER2 biparatopic IgG1 antibody with the microtubule inhibitor auristatin to construct an ADC. However, ZW49 and related studies such as MEDI4276 have been terminated due to toxicity and other reasons. Therefore, given the current gap in the dual-target ADC field, researchers could further consider methods to reduce ADC toxicity and attempt the construction of multi-target ADCs.

#### Small molecule drugs

Small molecule targeted therapies play a crucial role in GI cancer treatment by precisely targeting driver gene mutations or abnormal signaling pathways, effectively inhibiting tumor cell proliferation and growth. Regorafenib, sorafenib, and lenvatinib are FDA-approved small molecule drugs for GI cancers. Regorafenib demonstrated efficacy in refractory metastatic colorectal cancer patients in the CORRECT trial [318, 319]. Sorafenib, the first targeted drug approved for advanced liver cancer, significantly extended OS in the SHARP trial (NCT00105443) [320]. Similar to VEGF-targeting monoclonal antibodies, lenvatinib inhibits angiogenesis and tumor growth [321], showing comparable efficacy to sorafenib in advanced liver cancer [322]. Furthermore, PARP inhibitors are a class of therapeutic agents that primarily target and inhibit PARP1/2 proteins, thereby inducing “synthetic lethality” in tumor cells harboring *BRCA1/2* mutations or exhibiting HRD [323]. For gBRCA mutated metastatic PC, the PARP inhibitor Olaparib extended PFS by approximately four months compared to the placebo group in the POLO trial [45].

Significant association between *FGFR2* alteration and cholangiocarcinoma indicates the therapy pathway of FGFR. FDA-approved FGFR2 inhibitors for treating CCA include Pemigatinib, Infigratinib and Futibatinib [324–327]. Pemigatinib targets FGFR2 and achieved ORR in 35.5% of patients with *FGFR2* fusions or rearrangements [321]. Infigratinib and Futibatinib achieved ORRs of 23.1% and 41.7% respectively. With similar effects, among IDH inhibitors for gastrointestinal tumors, Ivosidenib is currently the only FDA-approved IDH inhibitor for treating previously treated locally advanced or metastatic CCA, targeting *IDH1* mutation. These drugs provide critical treatment options for GI cancer patients through multi-target precision inhibition.

In addition, numerous small molecule targeted drugs that have entered clinical trials have demonstrated significant therapeutic potential. Cabozantinib extended OS by approximately four months in advanced liver cancer patients in the CELESTIAL trial and is also applicable to CRC [328, 329]. However, in *FGFR2* amplified tumors (GC and gastroesophageal carcinoma), AZD4547 did not significantly improve PFS versus paclitaxel [330] [331]. Regarding *IDH1* mutations in CCA, the main drugs in clinical trial phases are LY3410738 and BAY1436032, both currently in phase I clinical stages, enrolling patients with *IDH1* mutation-positive advanced CAA [332, 333]. For mCRC, onartuzumab, a MET inhibitor, although phase II clinical trials have demonstrated its efficacy in combination with FOLFOX plus Bevacizumab, it is currently mainly used for non-small cell lung cancer (NSCLC) and has no further clinical trials related to gastrointestinal tumors [334]. Similarly, as a MET inhibitor, Savolitinib has received NMPA approval for treating NSCLC, but for gastrointestinal tumors (mainly GC and PC), it remains in the phase I clinical stage exploring pharmacokinetic and related properties [335, 336]. Selpercatinib targets *RET* fusion tumors, showing early efficacy in the LIBRETTO-001 trial [337].

Furthermore, *RAS* mutations are widely present in gastrointestinal tumors, with approximately 40.4% of metastatic colorectal cancer patients having *RAS* gene mutations [338], and related targeted drugs are generally in preclinical and clinical trial stages. This pan-RAS inhibitor ADT-007 selectively targets *RAS*-mutant cancer cells, demonstrates robust antitumor activity in CRC and PC models by suppressing MAPK signaling and activating the tumor immune microenvironment, while effectively circumventing resistance mechanisms associated with conventional RAS inhibitors [339]. KRAS G12D inhibitors in preclinical experiments demonstrate significant therapeutic potential by selectively targeting the mutant protein, showing robust antitumor activity including tumor regression in preclinical models, thereby offering a promising treatment strategy for patients with *KRAS* G12D-driven cancers that currently lack effective targeted therapies [340, 341]. Based on these promising preclinical data, a series of clinical trials targeting RAS inhibitors have been initiated and have preliminarily demonstrated their therapeutic potential. Pan-RAS inhibitors mainly include RMC-6236, targeting PDAC and JYP0015 (NCT06895031) targeting PDAC and CRC [342]. Focusing on *KRAS* G12D, GFH375 (VS-7375) has received fast track designation (FTD) for first-line treatment of patients with *KRAS* G12D mutation-positive locally advanced or metastatic pancreatic ductal adenocarcinoma, but is currently in phase I/II clinical study (NCT06500676) [343]. Additionally, INCB161734’s clinical research on various gastrointestinal tumors is also in the preliminary stage (NCT06500676). These drugs, through targeting specific molecular mechanisms and combination therapy strategies, provide innovative directions for GI cancer treatment and show potential for multi-indication development.

### Immunotherapy approaches

Immunotherapy is a therapeutic strategy designed to modulate or enhance the function of the immune system to recognize and eliminate tumor cells. The core concept is to utilize the body’s natural immune defense mechanisms, targeting tumor-specific antigens or immune-suppressive factors within the TME, to restore or strengthen anti-tumor immune responses and achieve therapeutic effects. Here we focus on immune checkpoint inhibitors (ICIs), tumor vaccines, and adoptive cell therapy, systematically analyzing their applications and developments in GI cancers.

#### Immune checkpoint inhibitors

Immune checkpoint inhibitors represent a major breakthrough in tumor immunotherapy in recent years [344]. These inhibitors mainly target immune checkpoint molecules, which under normal physiological conditions maintain immune self-tolerance and prevent excessive immune reactions. However, in the TME, these molecules are often exploited by tumors to evade immune surveillance [345, 346].

PD-1/PD-L1 and CTLA-4 are two well-studied immune checkpoints, and many ICIs targeting them have been approved for clinical use in GI cancers. For PD-1/PD-L1 inhibitors, trials such as KEYNOTE-016 have shown that pembrolizumab significantly improved PFS by approximately eight months compared to chemotherapy in MSI-H/dMMR mCRC, GC, and gastroesophageal junction cancer [347–350]. Additionally, nivolumab extended disease-free survival by nearly one year in esophageal cancer patients, with a lower incidence of adverse events compared to chemotherapy [351]. CTLA-4 inhibitors, such as ipilimumab, when combined with PD-1 inhibitors, further improved response rates in advanced colorectal cancer [352]. Other ICIs, such as atezolizumab (Tecentriq) combined with bevacizumab (Avastin), and durvalumab (Imfinzi), have been approved for hepatocellular carcinoma and CCA [282, 353].

Emerging ICIs in clinical trials offer new possibilities for GI cancer treatment, targeting traditional molecules like PD-1/PD-L1 and CTLA-4, as well as novel targets like LAG-3, TIGIT, and TIM-3. For example, relatlimab combined with nivolumab improved the 2-year recurrence-free survival (RFS) and OS rates in esophageal cancer patients to 72.5% and 82.6%, respectively [354, 355]. TIGIT inhibitors, such as tiragolumab, combined with atezolizumab and bevacizumab, achieved a 43% objective response rate in liver cancer [356]. Sabatolimab, a TIM-3 inhibitor, combined with spartalizumab showed preliminary anti-tumor activity [357]. Tremelimumab, a CTLA-4 inhibitor, combined with durvalumab has been studied for unresectable hepatocellular carcinoma [358]. The development of these novel ICIs further expands the possibilities for treating GI cancers and advances precision immunotherapy (Table 1).

**Table 1:** Targeted and immunotherapeutic agents in GI cancers

Cancer Type	Drugs	Mechanism	Indications	Clinical Trial Status	References
ESOPHAGEAL CANCER	Pembrolizumab	Specific PD-1/PD-L1 antibody	Advanced ESCC (PD-L1^+^ or MSI-H)	FDA-approved	[359]
	Nivolumab			Phase III NCT03143153	[360]
	Sintilimab			Phase II NCT03985046	[361]
	Trastuzumab	Specific HER2 antibody	Advanced or metastatic HER2-positive G/GEJ cancer	Phase II NCT04908813, NCT05504720	[362, 363]
GASTRIC/GEJ CANCER	Ramucirumab	Specific VEGFR2 antibody	Advanced HER2-negative GC or GEJC	Phase III NCT02934464	[364]
	Apatinib	Specific VEGFR2 small molecule inhibitor	Advanced GC third-line treatment	Phase IV NCT02426034	[365, 366]
	Nivolumab	Specific PD-1 antibody	MSI-H/dMMR or PD-L1^+^ advanced GC	Phase III NCT02872116	[367]
	Pembrolizumab			FDA-approved	[368, 369]
	Sintilimab			Phase II NCT03382600	[370]
	Zolbetuximab	Specific Claudin 18.2 antibody	Claudin18.2-positive GC first-line treatment	FDA-approved	[285, 371]
	Bemarituzumab	Specific FGFR2b antibody	FGFR2b-positive GC	Phase II NCT03694522	[372]
COLORECTAL CANCER	Cetuximab	Specific EGFR antibody	*RAS/BRAF* wild-type metastatic CRC	Phase I/II NCT00079066	[373]
	Panitumumab		*RAS/BRAF* wild-type metastatic CRC	Phase III NCT01412957	[374]
	Bevacizumab	Specific VEGF antibody	Metastatic CRC first/second-line treatment	Phase III NCT03950154	[1, 375]
	Regorafenib	Specific VEGFR, PDGFR, FGFR small molecule inhibitor	Metastatic CRC	Phase III NCT01103323	[318]
	Fruquintinib			Phase III NCT04322539	[376]
	Pembrolizumab	Specific PD-1 antibody	MSI-H/dMMR metastatic CRC	FDA-approved	[347]
	Nivolumab			Phase II NCT02060188	[352]
	Larotrectinib	Specific TRK small molecule inhibitor	*NTRK* fusion-positive solid tumors	Phase II NCT02122913, NCT02637687, NCT02576431	[8]
	Entrectinib			Phase I/II NCT02097810, NCT02568267	[377]
	Sotorasib	Specific *KRAS* G12C small molecule inhibitor	*KRAS* G12C mutant metastatic CRC	Phase I NCT03600883	[378]
	Adagrasib			Phase I/II NCT03785249	[379]
HEPATOCELLULAR CARCINOMA	Sorafenib	Specific PDGFR, RAF, VEGFR small molecule inhibitor	Unresectable HCC first-line treatment	Phase III NCT00105443	[320]
	Lenvatinib			Phase III NCT01761266	[380]
	Regorafenib	Multi-targeted tyrosine kinase inhibition	HCC progressed after sorafenib treatment	Phase III NCT01774344	[381]
	Cabozantinib			Phase III NCT01908426	[382]
PANCREATIC CANCER	Olaparib	Inhibiting DNA repair, synthetic lethality effect	*BRCA1/2* mutant metastatic PC maintenance therapy	Phase III NCT02184195	[45]
	Rucaparib			Phase II NCT03140670	[383]
	Larotrectinib	Specific TRK small molecule inhibitor	*NTRK* fusion-positive PC	Phase II NCT02122913, NCT02637687, NCT02576431	[8]
	Entrectinib			Phase I/II NCT02097810, NCT02568267	[377]
	Pembrolizumab	Specific PD-1 antibody	MSI-H/dMMR PC (rare <1%)	FDA-approved	[6, 384]
	Sotorasib	Specific *KRAS* G12C small molecule inhibitor	*KRAS* G12C mutant PC (rare)	Phase III NCT03600883	[385]
BILIARY TRACT CANCER	Pemigatinib	Specific FGFR small molecule inhibitor	*FGFR2* fusion/rearrangement CCA (10-15%)	Phase II NCT02924376	[327]
	Futibatinib			Phase II NCT02052778	[326]
	Ivosidenib	Specific IDH1 small molecule inhibitor	*IDH1*-mutant CCA (10-20%)	Phase III NCT02989857	[386]
	Durvalumab	Specific PD-L1 antibody	Advanced biliary tract cancer first-line treatment	Phase II NCT03875235	[387]
NEUROENDOCRINE TUMORS	Lanreotide	Somatostatin analogue, inhibiting hormone secretion and tumor proliferation	Well-differentiated GEP-NET	Phase III NCT00353496	[388]
	Everolimus	Specific mTOR small molecule inhibitor	Progressive pancreatic NETs	Phase II NCT00510068	[389]
	Sunitinib	Specific PDGFR, VEGFR small molecule inhibitor		Phase I/II NCT00428597	[390]
	^177^Lu-DOTATATE	Somatostatin receptor-targeted radiotherapy	Somatostatin receptor-positive GEP-NET	Phase III NCT01578239	[391]

*CCA* Cholangiocarcinoma, *CRC* Colorectal cancer, *ESCC* Esophageal squamous cell carcinoma/adenocarcinoma, *GC* Gastric cancer, *GEJC* Gastroesophageal junction adenocarcinoma, *GEP-NET* Gastro-entero-pancreatic neuroendocrine tumor, *HCC* Hepatocellular carcinoma, *PC* Pancreatic cancer. Data sources—https://clinicaltrials.gov/

Furthermore, many bispecific antibodies targeting immune checkpoints have also entered clinical trial stages. For example, AZD7789, an Anti-PD-1 and Anti-TIM-3 Bispecific Antibody, is currently conducting Phase I/IIa studies for GC and gastroesophageal junction adenocarcinoma (GEJC). Catumaxomab, a bispecific monoclonal antibody targeting EpCAM and CD3, has demonstrated early efficacy in gastric cancer patients with ascites [392]. Furthermore, Rilvegostomig is currently the fastest-progressing PD-1/TIGIT bispecific antibody, having entered phase III clinical trials for gastric cancer (NCT06764875) and hepatocellular carcinoma (NCT06921785).

#### Adoptive cell therapy

Adoptive cell therapy (ACT) involves the genetic modification of patient- or donor-derived immune cells ex vivo, followed by reinfusion of these engineered cells into the patient to enable specific targeting and elimination of tumor cells.

Chimeric antigen receptor T-cell (CAR-T) therapy is a type of adoptive immune cell therapy that involves genetically modifying a patient’s T cells to specifically recognize and efficiently kill tumor cells. However, GI cancers face significant challenges in cell therapy due to the complex immune-suppressive environment (e.g., Tregs, MDSCs) and difficulties in target selection. Currently, HER2-CAR-T, CLDN18.2-CAR-T, and MSLN-CAR-T therapies are being explored in phase I clinical trials to assess their stability and combination strategies [393–395]. For example, the first randomized controlled trial targeting solid tumors showed that CLDN18.2-CAR-T therapy (satri-cel) significantly extended PFS in patients with advanced gastric cancer or gastroesophageal junction cancer (3.25 vs. 1.77 months), with manageable safety, supporting its potential as a third-line treatment option [396].

Engineered T cell receptor-T cell (TCR-T cell) therapy has demonstrated potential in the treatment of GI tumors. The ROSENBERG team reported interim results from a phase II clinical trial (NCT03412877) targeting metastatic colorectal cancer, showing a progression-free survival (PFS) of 4.6 months. These findings indicate that personalized neoantigen-based TCR-T cell therapy can induce tumor regression and exhibit certain durability [397]. Additionally, TCR-T cell therapy targeting *KRAS* mutant antigens has shown preliminary success in PC. For instance, specific TCR-T cell therapy targeting the *KRAS*-G12D mutant antigen achieved promising efficacy in a patient with metastatic PC, with a 72% reduction in tumor burden six months post-treatment [398]. Furthermore, new targets such as mesothelin in PC and *KRAS*-G12V mutant-specific TCR-T cell therapy are currently under clinical investigation [399, 400]

In HBV-associated hepatocellular carcinoma, TCR-T cell therapy has shown potential application prospects. Among two patients receiving HBV antigen-specific TCR-T cell therapy, one patient exhibited significant reduction in five out of six pulmonary metastatic lesions [401]. Subsequently, a team conducted a phase I clinical trial using HBV-specific TCR-T cells (LioCyx-M004) to treat advanced HBV-associated HCC, achieving a median overall survival of 33.1 months [402]. These results suggest that HBV-specific TCR-T cells possess robust antiviral activity and in vivo tolerance.

In summary, TCR-T cell therapy has shown promising clinical efficacy in GI tumors, particularly in treatments targeting CEA, *KRAS* mutations, and HBV antigens. However, its therapeutic outcomes require further optimization, and serious adverse events during treatment need to be addressed to advance the broader application of this therapy in GI cancers.

#### Tumor vaccines

Tumor vaccines refer to the use of carriers such as peptides, nucleic acids, adenoviruses, or dendritic cells (DCs) to deliver tumor-associated antigens or neoantigens, combined with immune adjuvants or innate immune activation signals, to activate and expand antigen-specific CD8^+^ cytotoxic T cells and CD4^+^ helper T cells (as well as associated B cells and memory immunity). Meanwhile, they reshape the tumor microenvironment (enhancing immune infiltration and reducing immune suppression) [403, 404].

DCs are professional antigen-presenting cells that play a crucial role in the initiation and regulation of adaptive immune responses. Currently, many DC-based therapies for GI cancers have entered in clinical trials (NCT03152565, NCT00868114, NCT04317248). For example, the WT1-targeted DC vaccine combined with chemotherapy significantly activated WT1-specific immune responses, facilitated conversion surgery in patients with unresectable pancreatic ductal adenocarcinoma (UR-PDAC), and notably improved long-term clinical outcomes [405].Additionally, DCs transfected with *MUC1* mRNA achieved a one-year survival rate of over 50% in advanced PC patients [406]. Modified with poxvirus vectors encoding CEA and MUC1 (PANVAC), a randomized phase II trial investigated the use of dendritic cells and observed improved survival outcomes compared to patients who did not receive PANVAC vaccine during the same period, although the differences were not statistically significant [407]. Another trial targeting CRC patients with liver metastases, with DC loaded with autologous tumor lysates, extended DFS by approximately 16 months [408]. For MSI-type CRC, a phase I/II clinical trial loading CEA onto DCs is currently underway (NCT01885702). Moreover, two clinical trials evaluating the efficacy of DC vaccines in preventing recurrence in hypermutated CRC patients after surgical resection (NCT03730948) and in stage IV CRC patients (NCT02919644). To conclude, DC therapy is still in its early stages, with future directions focusing on combination strategies (e.g., DC vaccines combined with chemotherapy or ICIs) and optimizing DC preparation techniques to address DC deficiencies in late-stage GI cancer patients.

Peptide/protein vaccines are the most extensively developed category for GI cancers, characterized by diverse targets and dependence on human leukocyte antigen (HLA) restriction and adjuvant optimization. Early traditional peptide vaccines targeting tumor-associated antigens like NY-ESO-1, MAGE-A3/426 [409], TTK [410], WT1 [411, 412], and KIF20A have shown relatively limited efficacy. For instance, the hTERT peptide GV1001 extended OS by only four months in the phase III TeloVac trial for PC, highlighting the limitations of single-target short peptides [413]. In contrast, multi-epitope vaccines have demonstrated advantages over single-epitope vaccines by eliciting broader and more robust immune responses [414–416]. For instance, PolyPEPI1018 is a multi-peptide vaccine containing 12 immunogenic epitopes, which, in combination with TAS-102 chemotherapy in advanced MSS mCRC patients, induced stronger vaccine-specific humoral and T cell immune responses, extending PFS by approximately 8 months. [417, 418]. Neoantigen vaccines are rapidly advancing, with clinical trials underway for colorectal cancer (NCT04486378), gastric cancer (NCT05192460, NCT05227378), and PC (NCT04799431, NCT05111353). Incorporating adenoviral and self-amplifying mRNA vectors encoding the top 20 predicted neoantigens, a personalized neoantigen vaccine regimen aims at induce tumor-specific CD8^+^ T cell responses and improve PFS in mCRC patients [416].

Regarding viral vector vaccines, the research team developed a CRC-specific vaccine, Ad5-GUCY2C-PADRE, by linking GUCY2C to a universal helper T-cell epitope (pan DR reactive epitope, PADRE) that enhances immune responses and loading it into an adenoviral vector. In a phase I clinical trial, this vaccine was shown to safely induce antigen-specific CD8^+^ cytotoxic T cells without generating autoimmune CD4^+^ T cells, demonstrating split tolerance to GUCY2C [419]. Additionally, the study found that the MVA-5T4 vaccine and low-dose cyclophosphamide could independently induce antitumor immune responses, significantly prolonging PFS (5.6 vs 2.4 months) and OS (20.0 vs 10.3 months) in patients with metastatic colorectal cancer, without severe toxic effects [420]. The VRP-CEA vaccine primarily induces CEA-specific humoral immunity, and long-term survivors have been observed in both stage III and IV colorectal cancer patients, suggesting the vaccine has the potential to prolong OS [421]. Therefore, various vaccines have demonstrated favorable immune responses and survival benefits in the treatment of colorectal cancer. The whole tumor cell-based vaccine is also a type of therapeutic vaccine prepared using intact tumor cells, which activates a polyclonal T-cell immune response by presenting the full antigenic profile [422]. For example, the PC GVAX vaccine combined with the attenuated *Listeria* vector CRS-207 showed immune activation and early survival signals in initial studies [423]. Similarly, the α-Gal epitope-modified whole-cell vaccine Algenpantucel-L did not significantly improve DFS/OS, highlighting the limitations of whole-cell strategies in the highly immune-suppressive PC microenvironment [424].

**Fig. 3:**
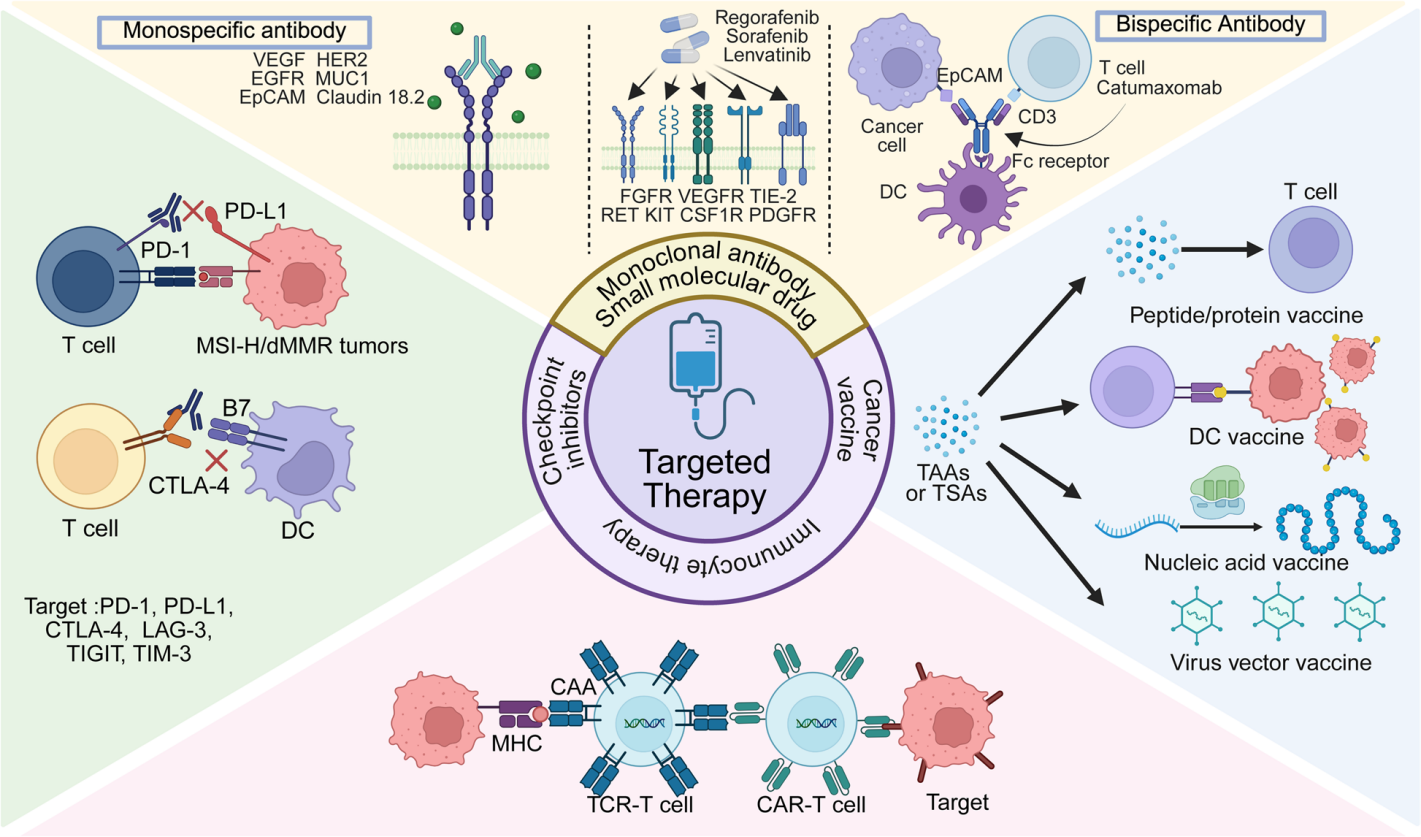
Overview of Mainstream Targeted Therapies for Gastrointestinal Cancer. Mainstream targeted therapeutic approaches for GI tumors can be divided into two major categories: monoclonal antibody and small molecular drugs, and immunotherapy. Monoclonal antibodies target molecules such as VEGF, HER2, EGFR, MUC1, EpCAM, and Claudin 18.2, while small molecule inhibitors block pathways involving FGFR, VEGFR, TIE-2, RET, KIT, CSF1R, and PDGFR. Immunotherapy comprises three key strategies: checkpoint inhibitors targeting PD-1, PD-L1, CTLA-4, LAG-3, TIGIT, and TIM-3 to enhance T cell responses, particularly in MSI-H/dMMR tumors; tumor vaccines, which utilize TAAs or TSAs in forms such as peptide/protein vaccines, DC vaccines, nucleic acid vaccines, and virus vector vaccines to stimulate anti-tumor immunity; and adoptive cell therapies, such as TCR-T and CAR-T cells, which target cancer-specific antigens or neoantigens to mediate direct tumor cell killing (figure was created with Biorender.com)

From an immunotherapy perspective, these approaches complement each other. PD-1/PD-L1 and CTLA-4 inhibitors provide a reliable foundation for immunotherapy, while immune cell therapy and tumor vaccines expand the range of immunotherapeutic targets. Together, these advances drive the diversification and precision of immunotherapy for GI cancers, while highlighting future opportunities to optimize treatment strategies and address challenges (Fig. 3).

With the in-depth investigation into the molecular mechanisms of tumorigenesis and progression, the application of relevant targeted drugs has demonstrated significant therapeutic efficacy advantages in clinical treatment. Various targeted agents, such as cetuximab, panitumumab, and regorafenib, have received FDA approval. However, clinical practice still faces challenges including drug resistance, off-target toxicity, and tumor heterogeneity. To address these issues, developing next-generation drugs such as multi-targeting bispecific antibodies (e.g., bispecific antibodies targeting HER2/HER3), combining different therapies like immunotherapy, and identifying effective biomarkers represent potential strategic solutions.

## Emerging therapies and future directions

### Mesenchymal stem cell-derived exosomes

**Fig. 4:**
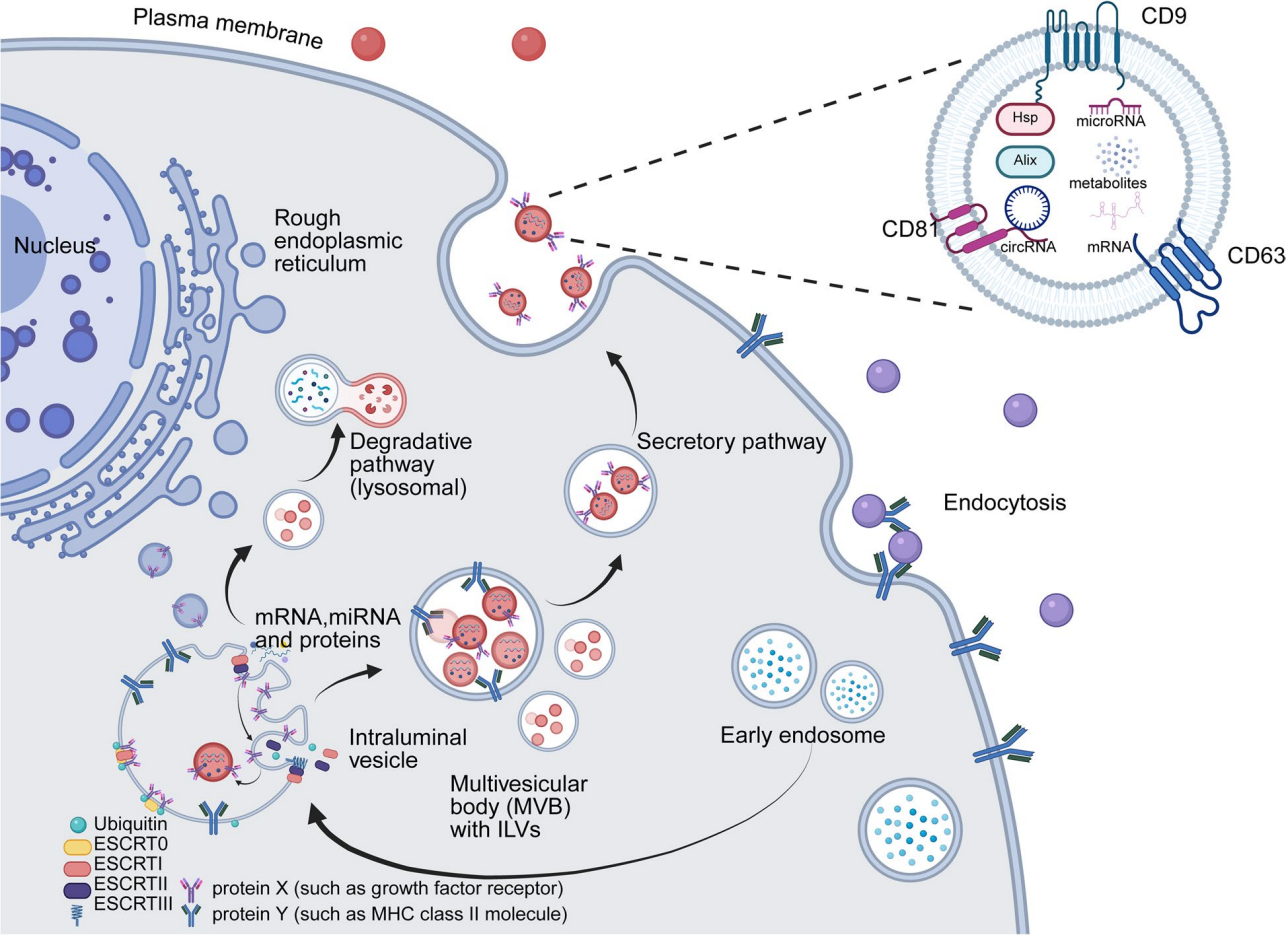
Biogenesis of MSC-Derived Exosomes in Emerging Targeted Therapies for Gastrointestinal Cancer. Exosome formation begins with endocytosis, where extracellular components are internalized into early endosomes. These endosomes mature into multivesicular bodies (MVBs) containing intraluminal vesicles (ILVs). ILV formation is mediated by the ESCRT (Endosomal Sorting Complex Required for Transport) machinery, which includes ESCRT-I, ESCRT-II, and ESCRT-III, along with ubiquitin and associated proteins (e.g., growth factor receptors or MHC class II molecules) (figure was created with Biorender.com)

Mesenchymal stem cells (MSCs), known for their regenerative and immunomodulatory properties, have emerged as key players in the TME, influencing the progression of various GI cancers (Fig. 4). MSC-derived exosomes have emerged as promising tools in the diagnosis and treatment of GI cancers due to their natural role in intercellular communication, biocompatibility, and ability to be engineered for targeted therapy. These nano-sized vesicles carry bioactive molecules such as proteins, lipids, and various forms of RNA, including microRNAs (miRNAs) and circular RNAs (circRNAs), which can modulate tumor behavior and the TME.

In GI cancers, MSC-derived exosomes exhibit dual roles: they can either promote or suppress tumor progression depending on their cargo. For instance, exosomal miR-221 from bone marrow MSCs enhances gastric cancer cell proliferation by inhibiting PI3K/AKT pathway suppressors [425], while exosomes carrying miR-199a-3p suppress HCC migration by targeting integrins [426]. Similarly, circ_0037104 from human MSC exosomes inhibits CCA by sponging miR-620 and upregulating APAF1 [427].

Therapeutically, MSC-derived exosomes are being harnessed as drug delivery vehicles due to their low immunogenicity and high targeting efficiency. They can be loaded with chemotherapeutic agents (e.g., paclitaxel, doxorubicin), nucleic acids (siRNA, miRNA mimics), or antisense oligonucleotides to enhance drug accumulation in tumors and reduce off-target effects [428]. For example, exosomes loaded with miR-122 sensitize HCC cells to sorafenib [429], and those carrying galectin-9 siRNA enhance immunotherapy in pancreatic ductal adenocarcinoma [430].

Moreover, exosomes influence the TME by modulating immune responses, angiogenesis, and extracellular matrix remodeling. They can promote immunosuppression via regulatory T cells or enhance anti-tumor immunity by activating natural killer cells [431]. Current clinical trials are exploring exosome-based diagnostics and therapies, though challenges in production scalability, cargo loading efficiency, and functional heterogeneity remain.

In conclusion, MSC-derived exosomes represent a versatile platform for GI cancer therapy, offering opportunities for precision medicine through targeted delivery and immunomodulation. Future research should focus on standardizing isolation techniques, optimizing engineering strategies, and validating efficacy in clinical settings.

### Personalized medicine and genomic approaches

The Cancer Genome Atlas has conducted large-scale genomic analyses to classify gastric cancer into four molecular subtypes: EBV-positive, MSI, GS, and CIN, providing a theoretical foundation for personalized treatment [432, 433]. EBV-positive gastric cancer is characterized by PD-L1/PD-L2 amplification and *PIK3CA* mutations, making it potentially sensitive to immunotherapy [434–436]. MSI tumors exhibit high tumor mutation burden (TMB) and are often associated with mismatch repair gene mutations, showing strong responses to PD-1/PD-L1 immune checkpoint inhibitors [437, 438]. GS subtype is linked to diffuse gastric cancer and commonly features *CDH1* mutations, while CIN subtype is associated with intestinal gastric cancer, frequently showing *TP53* mutations and *HER2* amplification [437, 439]. Additionally, TCGA research has revealed key genomic alterations in colorectal cancer, including frequent mutations in *APC*, *KRAS*, and *TP53*, as well as pathway disruptions in WNT and TGF-β signaling, driving advancements in targeted therapies [440]. In the field of immunotherapy, MSI-H or dMMR GI cancers have demonstrated remarkable efficacy with immune checkpoint inhibitors like Pembrolizumab, establishing a foundational biomarker for broad-spectrum cancer treatment independent of tumor type [441]. For HER2-positive gastric cancer, the landmark ToGA trial showed that combining Trastuzumab with chemotherapy significantly improved survival rates, setting a precedent for targeted therapy in gastric cancer [3]. Also, the development of drugs targeting specific mutations in the MAPK/ERK pathway, such as BRAF V600E, is a clear demonstration of the power of personalized medicine [442, 443]. Furthermore, the development and clinical application of liquid biopsy techniques, such as ctDNA detection, have enabled non-invasive monitoring, allowing for early diagnosis and real-time tracking of tumor genomic changes to guide treatment adjustment.

### Combination therapies

Combination therapy for GI cancers is built upon the principles of molecular subtyping, targeted therapy, immunotherapy, and treatment timing, aiming to enhance efficacy and overcome resistance [444]. In targeted therapy, studies on *KRAS* G12C-mutated colorectal cancer have demonstrated significant efficacy of Sotorasib (AMG 510) as a monotherapy, while combining it with anti-EGFR agents like Panitumumab is being explored to address resistance and improve outcomes [445–447]. Similarly, the BEACON CRC trial established the superiority of Encorafenib combined with Cetuximab over standard chemotherapy for *BRAF* V600E-mutated colorectal cancer [448–450]. Moreover, the BREAKWATER trial also demonstrated that encorafenib plus cetuximab (EC) with mFOLFOX6 extended PFS by 5 months compared to standard care [451]. These findings align with the rationale behind *KRAS*-targeted combinations, suggesting that targeting signaling pathways for specific molecular subtypes may be broadly effective. However, limitations remain, such as unclear efficacy of KRAS inhibitors in non-G12C mutations and challenges in optimizing safety and tolerability in combination regimens [452, 453]. Anti-angiogenic therapies, such as Bevacizumab combined with chemotherapy [454], have become the standard first-line treatment for metastatic colorectal cancer, while Ramucirumab has demonstrated survival benefits as a second-line option in advanced gastric cancer [455–457]. These studies collectively reinforce the critical role of VEGF pathway inhibition in advanced GI cancers, though its efficacy is constrained by the complexity of the TME, which invites further exploration of combinations with immunotherapies.

In immunotherapy, pivotal trials like Le et al.’s study and the CheckMate-649 trial have confirmed the substantial efficacy of combining use of immune checkpoint inhibitors (e.g., Pembrolizumab and Nivolumab) as first-line therapies in advanced gastric, gastro-oesophageal junction, and oesophageal adenocarcinoma [458, 459]. As well, the LEAP-015 trail of combining Lenvatinib and Pembrolizumab plus chrmotherapy confirms the manageable safety profile in the first-line treatment in advanced/metastatic gastroesophageal adenocarcinoma [460]. These findings are consistent with the success of immunotherapy in other malignancies, establishing its central role in specific molecular subtypes of GI cancers. However, immunotherapy has shown limited efficacy in non-MSI-H patients, and resistance mechanisms such as immunosuppressive factors in the TME (e.g., TGF-β) remain significant challenges [461]. This highlights the potential of combining immune checkpoint inhibitors with anti-angiogenic agents or TGF-β inhibitors to enhance therapeutic outcomes. Molecular subtyping plays a critical role in guiding combination therapy, as evidenced by the use of Trastuzumab combined with chemotherapy as the standard treatment for HER2-positive gastric cancer, as well as emerging strategies targeting *BRAF* mutations, *KRAS* mutations, and MSI-H/dMMR status [462, 463]. These studies collectively emphasize the importance of molecular subtyping in precision medicine (Table 2).

**Table 2:** Combination therapies targeting key molecular pathways in GI cancers

Therapy	Target	Indication	Clinical Trial Status	Reference
Pembrolizumab + Trastuzumab + Fluoropyrimidine and Platinum-based Chemotherapy	HER2, PD-1	HER2-positive GEJC (with PD-L1 CPS ≥ 1)	FDA-approved	[464]
Encorafenib + Cetuximab + mFOLFOX6	BRAF V600E	BRAF V600E-mutated CRC	FDA-approved	[448, 451, 465]
Nivolumab + Chemotherapy	PD-1	Advanced GC/GEJC	FDA-approved	[458]
Pembrolizumab + Chemotherapy	PD-1	Locally advanced or metastatic HER2-GC/GEJC	FDA-approved	[466]
Zolbetuximab + Chemotherapy	CLDN18.2	CLDN18.2-positive, HER2- GC/GEJC	FDA-approved	[36, 467]
Ramucirumab + FOLFIRI	VEGF2	Advanced GC/GEJC	FDA-approved	[468]
HLX22 + Trastuzumab + XELOX	HER2	HER2^+^ GC/GEJC	Phase III NCT04908813	[469]
Cadonilimab + XELOX	PD-1/CTLA-4	Locally advanced recurrent or metastatic GC/GEJC	Phase III NCT05008783	[470]
Lenvatinib + Pembrolizumab	HCC	Advanced HCC	Phase III NCT03713593	[471]
Durvalumab + FLOT Chemotherapy (Fluorouracil, Leucovorin, Oxaliplatin, Docetaxel)	PD-L1	GC/GEJC	Phase III NCT04592913	[472]
Lenvatinib + Pembrolizumab plus Chemotherapy	VEGF, PD-1	Advanced/Metastatic GEJC	Phase III NCT03713593	[460]

*CRC* Colorectal cancer, *GEJC* Gastroesophageal junction adenocarcinoma, *GC* Gastric cancer, *HCC* Hepatocellular carcinoma. Data sources—https://clinicaltrials.gov/

However, gaps remain in understanding treatment timing and sequencing strategies. For instance, the integration of immunotherapy and targeted therapy in neoadjuvant settings, as well as optimizing second-line combinations to extend survival, warrant further investigation. Overall, combination therapy for GI cancers leverages complementary mechanisms, synergistic effects, and molecular subtyping to build a framework for personalized treatment. Nevertheless, addressing limitations such as resistance, restricted efficacy, and unanswered questions about sequencing strategies is essential to broaden clinical applications and improve patient outcomes.

Despite the remarkable clinical success of targeted strategies for gastrointestinal cancers—including small-molecule inhibitors, monoclonal antibodies, and immunotherapy—their efficacy remains substantially limited by critical factors such as tumor heterogeneity, the evolution of drug resistance, and the immunosuppressive tumor microenvironment. These challenges are driving the exploration of novel therapeutic approaches, such as multi‑mechanism combination strategies. Molecular classification based on genomic profiling, which integrates multi‑omics data with clinicopathological features, offers a highly promising avenue for guiding more precise treatment. However, realizing its full potential requires a deeper molecular‑level understanding to identify robust biomarkers, which is essential for advancing and ultimately transforming the treatment paradigm for GI cancers.

## Conclusions

GI cancers represent a major global public health burden, with approximately 4.8 million new cases annually (in 2020) [473], among which colorectal cancer ranks third in incidence with an ascending trend [474]. The molecular pathogenesis of these malignancies exhibits remarkable complexity, encompassing multiple molecular aberrations including *KRAS* (exons 2, 3, and 4)/*NRAS* (exons 2, 3, and 4) mutations (40–50%) [475, 476], *PIK3CA* activation, p53/*APC* inactivation, MSI-H(15%) [477], alongside dysregulation of critical signaling pathways such as Wnt/β-catenin, PI3K/AKT/mTOR, and EGFR/HER2. Building upon comprehensive understanding of these molecular mechanisms, precision-targeted therapies have achieved significant breakthroughs, including anti-EGFR monoclonal antibodies for RAS wild-type patients [478, 479], anti-HER2 therapy for HER2-positive gastric cancer [480], and PD-1/PD-L1 inhibitors for MSI-H/dMMR patients, demonstrating objective response rates of 40–60% and offering renewed therapeutic hope for patients [481].

Looking forward, precision medicine for GI cancers is poised for revolutionary transformation. Liquid biopsy technologies (ctDNA, circulating tumor cells, exosomal miRNAs) will enable ultra-early diagnosis with anticipated sensitivity exceeding 90% [482]; artificial intelligence-driven multi-omics analysis will establish precise prognostic prediction models, facilitating truly personalized “one-patient-one-regimen” therapeutic approaches [483]; next-generation treatment strategies including bispecific antibodies, ADCs and proteolysis-targeting chimeras (PROTACs) will target previously “undruggable” molecular targets [300, 484, 485]; concurrently, TME reprogramming [486], precision nanomedicine delivery systems [487], and rationally designed combination therapies based on molecular network analysis will substantially enhance therapeutic efficacy while minimizing adverse effects. Within the next 5-10 years, treatment strategies tailored through comprehensive molecular profiling are expected to become standard clinical practice.

In summary, this article systematically reviews current research advances in the molecular pathogenesis of GI cancers from multiple perspectives, including genetic alterations, epigenetic regulation, and the tumor microenvironment. These mechanisms are intricately interconnected, collectively forming a sophisticated signaling regulatory network. Decades of in-depth analysis of this network have led to the development of numerous targeted drugs, which have achieved remarkable therapeutic outcomes in clinical practice. However, it must be emphasized that extensive crosstalk and compensatory mechanisms among different signaling pathways potentially confer tumor resistance to targeted therapies. In response, combination strategies based on multi-mechanism synergy, along with genomics-guided personalized and precision medicine, are emerging as critical future directions. By systematically synthesizing research progress on molecular mechanisms in gastrointestinal tumors and the current state of clinical translation of targeted drugs, as well as exploring promising emerging therapeutic strategies, this review aims to provide researchers with a more comprehensive and integrated perspective, thereby collectively advancing the development of targeted therapies and other innovative treatments for GI cancers.

## Data Availability

Not applicable.
